# Design, Synthesis and Bioactivities of Novel Dichloro-Allyloxy-Phenol-Containing Pyrazole Oxime Derivatives

**DOI:** 10.3390/molecules201219811

**Published:** 2015-12-08

**Authors:** Hong Dai, Linyu Ye, Huiyang Zhuang, Baojiang Dai, Yuan Fang, Yujun Shi

**Affiliations:** 1 College of Chemistry and Chemical Engineering, Nantong University, Nantong 226019, China; dh123@ntu.edu.cn (H.D.); lyuye265@aliyun.com (L.Y.); fyuan6586@aliyun.com (Y.F.); 2 Nantong Nanshen Plant Protection Science and Technology Development Co. Ltd., Nantong 226017, China; hyang6608@aliyun.com; 3 School of Chemical and Biological Engineering, Nantong Vocational University, Nantong 226007, China

**Keywords:** pyrazole oxime, dichloro-allyoxy-phenol, synthesis, biological activity

## Abstract

In this study, in order to find novel biologically active pyrazole oxime compounds, a number of dichloro-allyloxy-phenol-containing pyrazole oximes were designed and synthesized according to the method of active group combination. All of the target compounds were confirmed by ^1^H-NMR, ^13^C-NMR and elemental analysis. In addition, bioassays showed that all of the newly synthesized compounds had no acaricidal activity against *Tetranychus cinnabarinus* and low insecticidal activity against *Aphis craccivora* at tested concentrations. However, most of them displayed excellent insecticidal activity against *Oriental armyworm* at a concentration of 500 μg/mL, and some designed compounds still exhibited potent insecticidal activity against *Oriental armyworm* even at the dose of 20 μg/mL, especially compounds **7f**, **7n** and **7p** had 100%, 90% and 90% inhibition rates, respectively, which were comparable to that of the control pyridalyl.

## 1. Introduction

**Figure 1:**
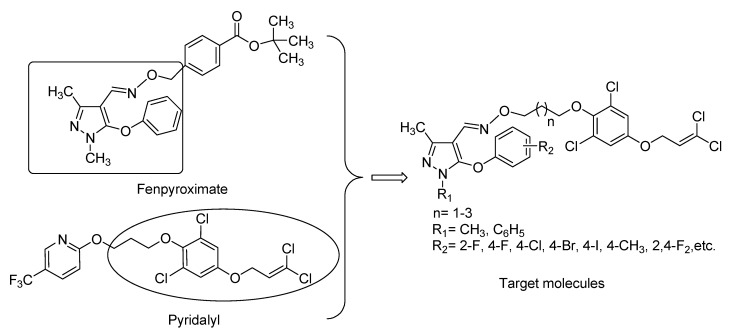
The design of the target molecules.

Pyrazole oximes are an important class of heterocyclic compounds which have drawn intense attention because of their good fungicidal [1,2], insecticidal [3,4,5], acaricidal [6], and anti-tobacco mosaic virus (TMV) activity [7]. For instance, Fenpyroximate (Figure 1), a potent agricultural acaricide with a vital pyrazole oxime backbone in the structure, possesses wonderful acaricidal property against some phytophagous mites such as *Polyphagotarsonemus latus* Banks and *Tetranychus urticae* Koch on different crops [8]. Since its appearance on the market in 1991, many chemists have begun to study structural modification of Fenpyroximate. More recently, Zou and co-workers reported some acaricidal and insecticidal Fenpyroximate analogues containing pyridine or thiazole group [9,10], and Wang *et al.* obtained some acaricidal, insecticidal or fungicidal pyrazole oxime compounds by replacing the substituted phenyl moiety of Fenpyroximate with oxazole ring [11]. Therefore, pyrazole oxime group can be used as an important skeleton in exploring novel bioactive molecules. 

Pyridalyl (Figure 1), a novel dichloro-allyloxy-phenol insecticide, was discovered by Sumitomo Chemical Co., Ltd. and marketed in 2004 [12]. It displays good insecticidal activity against different lepidopterous pests on various crops such as cotton and vegetables, and exhibits no cross-resistance with other existing insecticides including synthetic pyrethroids, organic phosphates, benzoylureas and nicotinic insecticides [13,14]. Its biochemical mode of action is still unknown, while recent study showed that pyridalyl can cause cell apoptosis in BM36 cells, and its insecticidal activities perhaps be related to the generation of active oxygen species of a pyridalyl metabolite [15]. In the past few years, a lot of biologically active dichloro-allyoxy-phenol-containing pyridalyl derivatives have been found [16,17,18,19]. It has been demonstrated that the dichloro-allyloxy-phenol group is essential for the activity of pyridalyl analogues [20,21].

Nowadays, owing to long-term use of traditional pesticides, several field populations of plant pests have already developed high levels of insecticide resistance [22], which leads to the great losses of crop production. Therefore, researchers have to search for novel, efficient, and low toxicity insecticides. In view on the facts above, the authors sought to incorporate the dichloro-allyloxy-phenol moiety into pyrazole oxime molecules by the intermediate derivatisation method [23]. In the present work, a series of novel dichloro-allyloxy-phenol-containing pyrazole oxime compounds were synthesized, and their acaricidal and insecticidal activities were screened briefly.

## 2. Results and Discussion

### 2.1. Chemistry

**Scheme 1:**
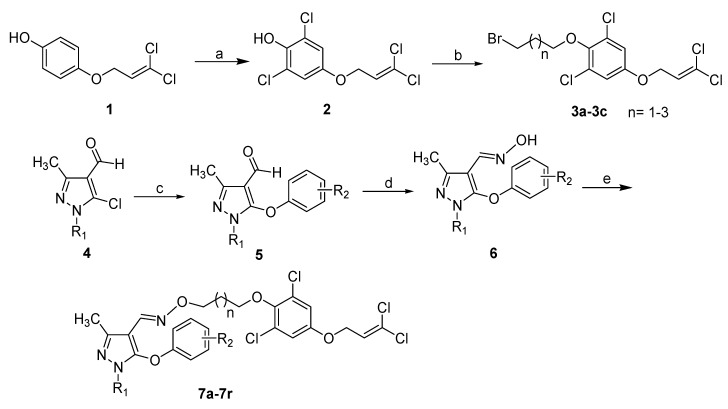
Synthesis of the title compounds **7a**–**7r**. *Reagents and conditions:* (a) sulfonyl chloride, toluene, diethylamine, 60–65 °C for 4 h, 60% for **2**; (b) BrCH_2_(CH_2_)nCH_2_Br, *n* = 1–3, potassium carbonate, *N*,*N*-dimethylformamide (DMF), 0 °C for 1 h, r.t. for 5–8 h, 46%–55% for **3**; (c) substituted phenols, potassium hydroxide, DMF or dimethylsulfoxide (DMSO), 45 °C for 2 h, then 110 °C for 5–24 h, 56%–78% for **5**; (d) hydroxylamine hydrochloride, potassium hydroxide, methanol or ethanol, reflux for 4–18 h, 61%–82% for **6**; (e) compounds **3a**–**3c**, potassium carbonate, acetonitrile, reflux for 10–24 h, 52%–71% for **7**.

As indicated in Scheme 1, 18 pyrazole oxime derivatives bearing dichloro-allyloxy-phenol moiety were successfully synthesized. Using diethylamine as the catalyst, 4-(3,3-dichloroallyloxy)-2,6-dichlorophenol (**2**) was obtained by the reaction of compound **1** with sulfonyl chloride in the solvent of toluene. Then intermediate **2** was reacted with dibromoalkanes in DMF with potassium carbonate as the base to form compound **3**. 5-aryloxy substituted pyrazole aldehyde **5** was produced by nucleophilic substitution of compound **4** with various substituted phenols under basic conditions. Subsequently, pyrazole aldehyde **5** was smoothly converted to pyrazole oxime **6** by treatment with hydroxylamine using potassium hydroxide as the base. Finally, the reaction of compound **6** with the key intermediate **3** under potassium carbonate promoting conditions afforded the title compounds **7a**–**7r** in good yields. The structures of all the target compounds were effectively determined by ^1^H-NMR, ^13^C-NMR and elemental analysis.

### 2.2. Biological Activities

The acaricidal activity against *Tetranychus cinnabarinus* and insecticidal activity and against *Oriental armyworm* and *Aphis craccivora* of all target compounds were tested and the data were listed in Table 1. Fenpyroximate, Pyridalyl and Imidacloprid were used as the positive controls, respectively. The linkage moiety between the pyrazole oxime and 1,1-dichloropropene were three (**7a**–**7m**), four (**7n**–**7p**), and five (**7q** and **7r**) carbon chains. As displayed in Table 1, all of the title compounds possessed no activities against *Tetranychus cinnabarinus* at a concentration of 500 μg/mL, and most of them showed lower activity against *Aphis craccivora* except compound **7q** had 70% inhibition rate at a dosage of 500 μg/mL. Encouragingly, most target compounds exhibited good larvicidal activity against *Oriental armyworm* at a concentration of 500 μg/mL, which was comparable to that of the control Pyridalyl. Moreover, some of the designed compounds displayed excellent larvicidal activity against *Oriental armyworm* when the concentration was reduced to 100 μg/mL. For example, compounds **7a**, **7b**, **7f**, **7k**, **7n**, **7o**, **7p**, and **7q** achieved 100% inhibition against *Oriental armyworm*. Among them, compounds **7f**, **7n**, and **7p** were still active against *Oriental armyworm* even when the dosage was reduced to 20 μg/mL with inhibitory values of 100%, 90%, and 90%, respectively, which were similar to that of the control Pyridalyl. 

From the data presented in Table 1, we found that the structure-activity relationships of the aimed compounds were not very obvious. However, fluorinated derivatives showed relatively better larvicidal activity against *Oriental armyworm* than other analogues. At the concentration of 20 μg/mL, compounds **7f**, **7n**, **7o**, **7p**, and **7q** had 100%, 90%, 60%, 90% and 50% inhibitory effects against *Oriental armyworm*, respectively. When the carbon chain that links the pyrazole oxime and dichloro-allyloxy-phenol moieties is three carbons, the 4-fluorosubstituted analogue **7f** exhibited a higher insecticidal activity against *Oriental armyworm* than did the corresponding 2- and 3-substituted analogues (**7d** and **7e**), and compounds **7d**, **7e**, and **7f** displayed 0%, 40%, and 100% larvicidal activity against *Oriental armyworm* at the concentration of 100 μg/mL, respectively. Based on the structure-potency data, we can also see that compound **7n** with a 1,4-butylenedioxy group is more active against *Oriental armyworm* than corresponding compound **7d** owning a 1,3-propylenedioxy group. When the enlargement of the linkage mioety between the dichloro-allyloxy-phenol and pyrazole oxime units is five carbons, compound **7q** (R_1_ = CH_3_, R_2_ = 4-F) exhibited better biological activities than compound **7r** (R_1_ = C_6_H_5_, R_2_ = 4-F), and compound **7q** displayed interesting insecticidal activity against *Aphis craccivora* beyond satisfactory larvicidal activity against *Oriental armyworm*. All the above data implied that structural modification of Fenpyroximate by dichloro-allyloxy-phenol moiety could give some new compounds possessing good biological activities. To obtain more active derivatives, further analogue synthesis and structural optimization are currently in progress.

**Table 1:** Insecticidal and acaricidal activities of compounds **7a**–**7r** (mortality, %).

Compd.	*n*	R_1_	R_2_	*Oriental armyworm*			*Aphis craccivora*		*Tetranychus cinnabarinus*
				500 μg/mL	100 μg/mL	20 μg/mL	500 μg/mL	100 μg/mL	500 μg/mL
**7a**	1	CH_3_	4-OCH_3_	100	100	0	0	—	0
**7b**	1	CH_3_	4-CH_3_	100	100	0	0	—	0
**7c**	1	CH_3_	4-OCF_3_	80	50	0	0	—	0
**7d**	1	CH_3_	2-F	100	0	—	0	—	0
**7e**	1	CH_3_	3-F	100	40	0	0	—	0
**7f**	1	CH_3_	4-F	100	100	100	0	—	0
**7g**	1	CH_3_	2-Cl	100	0	—	20	—	0
**7h**	1	CH_3_	4-Cl	100	0	—	0	—	0
**7i**	1	CH_3_	4-Br	0	—	—	0	—	0
**7j**	1	CH_3_	4-I	80	0	—	0	—	0
**7k**	1	CH_3_	3-NO_2_	100	100	0	20	—	0
**7l**	1	CH_3_	3-CF_3_	80	0	—	0	—	0
**7m**	1	CH_3_	2,4-Cl_2_	70	—	—	0	—	0
**7n**	2	CH_3_	2-F	100	100	90	0	—	0
**7o**	2	CH_3_	4-F	100	100	60	0	—	0
**7p**	2	CH_3_	2,4-F_2_	100	100	90	0	—	0
**7q**	3	CH_3_	4-F	100	100	50	70	20	0
**7r**	3	C_6_H_5_	4-F	—	—	—	0	—	0
Pyridalyl				100	100	100	—	—	—
Imidacloprid				—	—	—	100	100	—
Fenpyroximate				—	—	—	—	—	100

“—” refers to “not tested”.

## 3. Experimental Section

### 3.1. Chemistry

#### 3.1.1. General Procedures

All reagents were chemically pure and solvents were dried according to standard methods. ^1^H-NMR and ^13^C-NMR spectra were obtained on a Bruker AV400 spectrometer (400 MHz, ^1^H; 100 MHz, ^13^C, Bruker, Billerica, MA, USA) in CDCl_3_ with tetramethylsilane as the internal standard. The melting points were determined on an X-4 binocular microscope melting point apparatus (Beijing Tech Instrument Co., Beijing, China) and are uncorrected. Elemental analyses were determined on a Yanaco CHN Corder MT-3 elemental analyzer (Yanaco, Kyoto, Japan). The reactions were monitored by analytical thin-layer chromatography (TLC) with ultraviolet (UV) light and TLC was carried out on silica gel GF_254_. The intermediates 4-(3,3-dichloroallyloxy)phenol (**1**) and 4-(3,3-dichloroallyloxy)-2,6-dichlorophenol (**2**) were obtained by literature methods [24]. 5-chloropyrazole aldehyde **4** and 5-aryloxypyrazole aldehyde **5** were synthesized according to a reported procedure [25]. 

#### 3.1.2. General Procedure for the Preparation of **3a**–**3c**

To a stirred solution of intermediate **2** (20 mmol) in anhydrous DMF was added powered potassium carbonate (22 mmol) at room temperature, and the mixture was cooled to 0 °C. To the above mixture, was added dropwise a solution of various dibromoalkanes (20 mmol) in anhydrous DMF (15 mL). The resulting reaction mixture was stirred for another 50 min at 0 °C, and for 3–6 h at room temperature. The mixture was poured into water (150 mL) and extracted with ethyl acetate (3 × 50 mL). The organic layer was washed with saturated brine (3 × 30 mL) and dried over anhydrous MgSO_4_. The solvent was evaporated under reduced pressure, and the residue was subjected to silica gel column chromatography with petroleum ether and ethyl acetate as an eluent to afford the compounds **3a**–**3c**, with the yields ranging from 46% to 55% [19].

#### 3.1.3. General Procedure for the Preparation of **6a**–**6r**

To a stirred solution of hydroxylamine hydrochloride (15 mmol) in methanol or ethanol was added potassium hydroxide (20 mmol) in portions at room temperature. The mixture was stirred for another 20 min. To the above mixture, was added intermediate **5** (10 mmol) in portions. The resulting mixture was heated to reflux for 4–15 h. After cooling to room temperature, the reaction mixture was poured into water (100 mL) and allowed to stand overnight. The precipitate was collected by filtration and washed with water (3 × 50 mL). The solid residue was recrystallized from ethanol to produce the pyrazole oximes **6a**–**6r**, with yields ranging from 61% to 82% [11].

#### 3.1.4. General Procedure for the Preparation of **7a**–**7r**

To a well-stirred solution of pyrazole oxime **6** (10 mmol) and powered potassium carbonate (20 mmol) in anhydrous acetonitrile (25 mL) was added intermediate **3** (12 mmol) in one portion at room temperature. The mixture was then heated to reflux for 10–24 h. On completion, the mixture was poured into water (60 mL) and extracted with dichloromethane (3 × 40 mL). The combined organic layer was washed with 10% sodium carbonate solution (3 × 20 mL) and then with water (3 × 20 mL) and dried over anhydrous MgSO_4_. The solvent was removed under reduced pressure, and the residue was separated by silica gel column chromatography with petroleum ether and ethyl acetate as an eluent to afford the target compounds **7a**–**7r**, with yields ranging from 52% to 71%. All 18 pyrazole oxime derivatives **7a**–**7r** were novel and the physical and spectral data for these compounds are listed below. ^1^H-NMR and ^13^C-NMR spectra are provided in the Supplementary Materials.

*1-Methyl-3-methyl-5-(4-methyoxyphenoxy)-1H-pyrazole-4-carbaldehyde-O-{3-[2,6-dichloro-4-(3,3-dichloroallyoxy)-phenoxy]propyl}-oxime* (**7a**): Yellow oil, yield 69%. ^1^H-NMR (CDCl_3_): δ 7.76 (s, 1H, CH=N), 6.82–6.86 (m, 6H, Ar-H), 6.11 (t, *J* = 6.4 Hz, 1H, C=CH-CH_2_O), 4.58 (d, *J* = 6.0 Hz, 2H, C=CH-CH_2_O), 4.24 (t, *J* = 6.4 Hz, 2H, CH_2_O-Ar), 4.01 (t, *J* = 6.4 Hz, 2H, CH_2_O-N=CH), 3.77 (s, 3H, Ar-OCH_3_), 3.61 (s, 3H, N-CH_3_), 2.37 (s, 3H, CH_3_), 2.07–2.13 (m, 2H, CH_2_CH_2_CH_2_); ^13^C-NMR (CDCl_3_): δ 155.8, 153.9, 150.7, 148.3, 146.8, 146.0, 140.4, 129.8, 124.6, 119.8, 116.4, 115.2, 114.9, 100.0, 70.7, 70.4, 65.5, 55.7, 34.2, 29.7, 14.8. Anal. Calcd for C_25_H_25_Cl_4_N_3_O_5_: C 50.95; H 4.28; N 7.13. Found: C 50.82; H 4.46; N 7.30.

*1-Methyl-3-methyl-5-(4-methylphenoxy)-1H-pyrazole-4-carbaldehyde-O-{3-[2,6-dichloro-4-(3,3-dichloroallyoxy)-phenoxy]propyl}-oxime* (**7b**): Yellow oil, yield 71%. ^1^H-NMR (CDCl_3_): δ 7.77 (s, 1H, CH=N), 7.10 (d, *J* = 8.8 Hz, 2H, Ar-H), 6.83 (s, 2H, Ar-H), 6.78 (d, *J* = 8.8 Hz, 2H, Ar-H), 6.11 (t, *J* = 6.4 Hz, 1H, C=CH-CH_2_O), 4.58 (d, *J* = 6.0 Hz, 2H, C=CH-CH_2_O), 4.24 (t, *J* = 6.4 Hz, 2H, CH_2_O-Ar), 4.00 (t, *J* = 6.4 Hz, 2H, CH_2_O-N=CH), 3.59 (s, 3H, N-CH_3_), 2.37 (s, 3H, CH_3_), 2.30 (s, 3H, Ar-CH_3_), 2.06–2.12 (m, 2H, CH_2_CH_2_CH_2_); ^13^C-NMR (CDCl_3_): δ 154.7, 153.9, 148.0, 146.8, 146.0, 140.4, 133.1, 130.6, 130.4, 129.8, 124.6, 115.2, 115.1, 100.3, 70.7, 70.4, 65.5, 34.2, 29.7, 20.6, 14.8. Anal. Calcd for C_25_H_25_Cl_4_N_3_O_4_: C 52.38; H 4.40; N 7.33. Found: C 52.21; H 4.25; N 7.17.

*1-Methyl-3-methyl-5-(4-trifluoromethoxyphenoxy)-1H-pyrazole-4-carbaldehyde-O-{3-[2,6-dichloro-4-(3,3-dichloroallyoxy)-phenoxy]propyl}-oxime* (**7c**)*:* Yellow oil, yield 66%. ^1^H-NMR (CDCl_3_): δ 7.70 (s, 1H, CH=N), 7.09 (d, *J* = 8.8 Hz, 2H, Ar-H), 6.84 (d, *J* = 9.2 Hz, 2H, Ar-H), 6.75 (s, 2H, Ar-H), 6.03 (t, *J* = 6.4 Hz, 1H, C=C**H**-CH_2_O), 4.50 (d, *J* = 6.4 Hz, 2H, C=CH-C**H**_2_O), 4.12 (t, *J* = 6.4 Hz, 2H, CH_2_O-Ar), 3.91 (t, *J* = 6.4 Hz, 2H, C**H**_2_O-N=CH), 3.55 (s, 3H, N-CH_3_), 2.30 (s, 3H, CH_3_), 1.95–2.00 (m, 2H, CH_2_C**H**_2_CH_2_); ^13^C-NMR (CDCl_3_): δ 153.9, 148.7, 147.0, 146.3, 146.0, 139.6, 129.8, 128.1, 124.6, 124.0, 123.8, 115.5, 115.2, 100.5, 70.6, 70.5, 65.5, 34.1, 29.6, 14.5. Anal. Calcd for C_25_H_22_Cl_4_F_3_N_3_O_5_: C 46.68; H 3.45; N 6.53. Found: C 46.53; H 3.62; N 6.38.

*1-Methyl-3-methyl-5-(2-fluorophenoxy)-1H-pyrazole-4-carbaldehyde-O-{3-[2,6-dichloro-4-(3,3-dichloroallyoxy)-phenoxy]propyl}-oxime* (**7d**): Yellow oil, yield 52%. ^1^H-NMR (CDCl_3_): δ 7.77 (s, 1H, CH=N), 7.15–7.20 (m, 1H, Ar-H), 7.00–7.07 (m, 2H, Ar-H), 6.76–6.85 (m, 3H, Ar-H), 6.11 (t, *J* = 6.4 Hz, 1H, C=CH-CH_2_O), 4.58 (d, *J* = 6.0 Hz, 2H, C=CH-CH_2_O), 4.20 (t, *J* = 6.4 Hz, 2H, CH_2_O-Ar), 3.98 (t, *J* = 6.4 Hz, 2H, CH_2_O-N=CH), 3.67 (s, 3H, N-CH_3_), 2.36 (s, 3H, CH_3_), 2.03–2.09 (m, 2H, CH_2_CH_2_CH_2_); ^13^C-NMR (CDCl_3_): δ 153.9, 153.2, 152.0 (d, *J* = 247 Hz), 147.2, 147.0, 146.0, 144.3, 144.2, 139.8, 129.8, 125.0, 124.6, 124.4 (d, *J* = 7 Hz), 117.1 (d, *J* = 18 Hz), 116.7, 115.2, 100.0, 70.6, 70.5, 65.5, 34.2, 29.6, 14.5. Anal. Calcd for C_24_H_22_Cl_4_FN_3_O_4_: C 49.94; H 3.84; N 7.28. Found: C 49.82; H 4.03; N 7.45.

*1-Methyl-3-methyl-5-(3-fluorophenoxy)-1H-pyrazole-4-carbaldehyde-O-{3-[2,6-dichloro-4-(3,3-dichloroallyoxy)-phenoxy]propyl}-oxime* (**7e**)*:* Yellow oil, yield 54%. ^1^H-NMR (CDCl_3_): δ 7.78 (s, 1H, CH=N), 7.23–7.29 (m, 1H, Ar-H), 6.78–6.82 (m, 3H, Ar-H), 6.63–6.69 (m, 2H, Ar-H), 6.11 (t, *J* = 6.4 Hz, 1H, C=CH-CH_2_O), 4.57 (d, *J* = 6.4 Hz, 2H, C=CH-CH_2_O), 4.22 (t, *J* = 6.4 Hz, 2H, CH_2_O-Ar), 3.99 (t, *J* = 6.4 Hz, 2H, CH_2_O-N=CH), 3.62 (s, 3H, N-CH_3_), 2.38 (s, 3H, CH_3_), 2.04–2.11 (m, 2H, CH_2_CH_2_CH_2_); ^13^C-NMR (CDCl_3_): δ 158.7 (d, *J* = 241 Hz), 153.8, 152.6, 152.5, 147.5, 146.8, 145.9, 139.9, 129.7, 124.8, 124.5, 116.5, 116.4, 116.2, 115.1, 100.1, 70.5, 70.4, 65.4, 34.1, 29.6, 14.5. Anal. Calcd for C_24_H_22_Cl_4_FN_3_O_4_: C 49.94; H 3.84; N 7.28. Found: C 50.06; H 3.69; N 7.12.

*1-Methyl-3-methyl-5-(4-fluorophenoxy)-1H-pyrazole-4-carbaldehyde-O-{3-[2,6-dichloro-4-(3,3-dichloroallyoxy)-phenoxy]propyl}-oxime* (**7f**)*:* Yellow oil, yield 62%. ^1^H-NMR (CDCl_3_): δ 7.78 (s, 1H, CH=N), 6.86–7.04 (m, 4H, Ar-H), 6.85 (s, 2H, Ar-H), 6.13 (t, *J* = 6.0 Hz, 1H, C=CH-CH_2_O), 4.60 (d, *J* = 6.0 Hz, 2H, C=CH-CH_2_O), 4.24 (t, *J* = 6.0 Hz, 2H, CH_2_O-Ar), 4.01 (t, *J* = 6.0 Hz, 2H, CH_2_O-N=CH), 3.63 (s, 3H, N-CH_3_), 2.39 (s, 3H, CH_3_), 2.06–2.13 (m, 2H, CH_2_CH_2_CH_2_); ^13^C-NMR (CDCl_3_): δ 153.9, 146.9, 146.3 (d, *J* = 250 Hz), 146.0, 136.7, 129.8, 125.0, 124.6, 116.6, 116.3, 115.2, 100.2, 70.6, 70.5, 65.5, 34.2, 29.7, 14.6. Anal. Calcd for C_24_H_22_Cl_4_FN_3_O_4_: C 49.94; H 3.84; N 7.28. Found: C 50.12; H 3.72; N 7.21.

*1-Methyl-3-methyl-5-(2-chlorophenoxy)-1H-pyrazole-4-carbaldehyde-O-{3-[2,6-dichloro-4-(3,3-dichloroallyoxy)-phenoxy]propyl}-oxime* (**7g**)*:* Yellow oil, yield 53%. ^1^H-NMR (CDCl_3_): δ 7.75 (s, 1H, CH=N), 7.44–7.46 (m, 1H, Ar-H), 7.03–7.18 (m, 2H, Ar-H), 6.83 (s, 2H, Ar-H), 6.69–6.71 (m, 1H, Ar-H), 6.11 (t, *J* = 6.4 Hz, 1H, C=CH-CH_2_O), 4.58 (d, *J* = 6.0 Hz, 2H, C=CH-CH_2_O), 4.20 (t, *J* = 6.4 Hz, 2H, CH_2_O-Ar), 3.98 (t, *J* = 6.4 Hz, 2H, CH_2_O-N=CH), 3.65 (s, 3H, N-CH_3_), 2.37 (s, 3H, CH_3_), 2.03–2.09 (m, 2H, CH_2_CH_2_CH_2_); ^13^C-NMR (CDCl_3_): δ 153.9, 152.2, 147.1, 146.9, 146.0, 139.7, 130.9, 129.8, 128.0, 124.6, 124.5, 122.9, 121.2, 115.6, 115.2, 100.3, 70.6, 70.5, 65.5, 34.2, 29.7, 14.4. Anal. Calcd for C_24_H_22_Cl_5_N_3_O_4_: C 48.55; H 3.73; N 7.08. Found: C 48.36; H 3.90; N 7.19.

*1-Methyl-3-methyl-5-(4-chlorophenoxy)-1H-pyrazole-4-carbaldehyde-O-{3-[2,6-dichloro-4-(3,3-dichloroallyoxy)-phenoxy]propyl}-oxime* (**7h**)*:* Yellow oil, yield 61%. ^1^H-NMR (CDCl_3_): δ 7.77 (s, 1H, CH=N), 7.30 (d, *J* = 9.6 Hz, 2H, Ar-H), 6.86–6.93 (m, 4H, Ar-H), 6.14 (t, *J* = 6.0 Hz, 1H, C=CH-CH_2_O), 4.61 (d, *J* = 5.6 Hz, 2H, C=CH-CH_2_O), 3.97–4.25 (m, 4H, CH_2_O-Ar and CH_2_O-N=CH), 3.68 (s, 3H, N-CH_3_), 2.43 (s, 3H, CH_3_), 2.02–2.10 (m, 2H, CH_2_CH_2_CH_2_); ^13^C-NMR (CDCl_3_): δ 154.0, 152.9, 146.2, 145.8, 144.9, 138.5, 129.3, 128.9, 128.7, 123.9, 123.5, 118.2, 115.7, 114.2, 99.5, 69.6, 69.5, 64.5, 33.2, 28.6, 13.3. Anal. Calcd for C_24_H_22_Cl_5_N_3_O_4_: C 48.55; H 3.73; N 7.08. Found: C 48.71; H 3.55; N 6.92.

*1-Methyl-3-methyl-5-(4-bromophenoxy)-1H-pyrazole-4-carbaldehyde-O-{3-[2,6-dichloro-4-(3,3-dichloroallyoxy)-phenoxy]propyl}-oxime* (**7i**): Yellow oil, yield 65%. ^1^H-NMR (CDCl_3_): δ 7.79 (s, 1H, CH=N), 7.43 (d, *J* = 8.8 Hz, 2H, Ar-H), 6.80–6.85 (m, 4H, Ar-H), 6.14 (t, *J* = 6.4 Hz, 1H, C=CH-CH_2_O), 4.60 (d, *J* = 6.4 Hz, 2H, C=CH-CH_2_O), 4.24 (t, *J* = 6.4 Hz, 2H, CH_2_O-Ar), 4.01 (t, *J* = 6.4 Hz, 2H, CH_2_O-N=CH), 3.63 (s, 3H, N-CH_3_), 2.39 (s, 3H, CH_3_), 2.05–2.12 (m, 2H, CH_2_CH_2_CH_2_); ^13^C-NMR (CDCl_3_): δ 156.7, 155.8, 153.9, 147.0, 146.0, 139.9, 139.3, 132.8, 129.8, 125.0, 124.6, 122.4, 117.1, 116.1, 115.2, 100.4, 70.6, 70.5, 65.5, 34.2, 29.7, 14.5. Anal. Calcd for C_24_H_22_BrCl_4_N_3_O_4_: C 45.17; H 3.47; N 6.58. Found: C 45.06; H 3.63; N 6.45.

*1-Methyl-3-methyl-5-(4-iodophenoxy)-1H-pyrazole-4-carbaldehyde-O-{3-[2,6-dichloro-4-(3,3-dichloroallyoxy)-phenoxy]propyl}-oxime* (**7j**): Yellow oil, yield 67%. ^1^H-NMR (CDCl_3_): δ 7.76 (s, 1H, CH=N), 7.60 (d, *J* = 8.8 Hz, 2H, Ar-H), 6.83 (s, 2H, Ar-H), 6.68 (d, *J* = 8.8 Hz, 2H, Ar-H), 6.11 (t, *J* = 6.4 Hz, 1H, C=CH-CH_2_O), 4.58 (d, *J* = 6.4 Hz, 2H, C=CH-CH_2_O), 4.21 (t, *J* = 6.4 Hz, 2H, CH_2_O-Ar), 3.99 (t, *J* = 6.4 Hz, 2H, CH_2_O-N=CH), 3.61 (s, 3H, N-CH_3_), 2.37 (s, 3H, CH_3_), 2.03–2.09 (m, 2H, CH_2_CH_2_CH_2_); ^13^C-NMR (CDCl_3_): δ 156.6, 153.9, 147.0, 146.9, 146.0, 139.8, 139.2, 138.8, 129.8, 125.0, 124.6, 119.7, 117.6, 115.2, 100.4, 86.4, 70.7, 70.5, 65.5, 34.2, 29.7, 14.5. Anal. Calcd for C_24_H_22_Cl_4_IN_3_O_4_: C 42.07; H 3.24; N 6.13. Found: C 42.26; H 3.05; N 6.01.

*1-Methyl-3-methyl-5-(3-nitrophenoxy)-1H-pyrazole-4-carbaldehyde-O-{3-[2,6-dichloro-4-(3,3-dichloroallyoxy)-phenoxy]propyl}-oxime* (**7k**)*:* Yellow oil, yield 55%. ^1^H-NMR (CDCl_3_): δ 7.99 (d, *J* = 8.4 Hz, 1H, Ar-H), 7.80 (s, 1H, CH=N), 7.77 (s, 1H, Ar-H), 7.27–7.54 (m, 2H, Ar-H), 6.84 (s, 2H, Ar-H), 6.13 (t, *J* = 6.4 Hz, 1H, C=CH-CH_2_O), 4.60 (d, J = 6.4 Hz, 2H, C=CH-CH_2_O), 4.14 (t, *J* = 6.4 Hz, 2H, CH_2_O-Ar), 3.95 (t, *J* = 6.4 Hz, 2H, CH_2_O-N=CH), 3.68 (s, 3H, N-CH_3_), 2.39 (s, 3H, CH_3_), 1.97–2.03 (m, 2H, CH_2_CH_2_CH_2_); ^13^C-NMR (CDCl_3_): δ 157.0, 153.9, 149.3, 147.3, 145.6, 139.4, 130.6, 129.7, 125.0, 124.6, 121.6, 118.5, 115.2, 110.8, 100.4, 70.6, 70.4, 65.5, 34.3, 29.6, 14.1. Anal. Calcd for C_24_H_22_Cl_4_N_4_O_6_: C 47.70; H 3.67; N 9.27. Found: C 47.85; H 3.53; N 9.38.

*1-Methyl-3-methyl-5-(3-trifluoromethylphenoxy)-1H-pyrazole-4-carbaldehyde-O-{3-[2,6-dichloro-4-(3,3-dichloroallyoxy)-phenoxy]propyl}-oxime* (**7l**): Yellow oil, yield 57%. ^1^H-NMR (CDCl_3_): δ 7.80 (s, 1H, CH=N), 7.38–7.48 (m, 2H, Ar-H), 7.22 (s, 1H, Ar-H), 7.07 (d, *J* = 8.0 Hz, 1H, Ar-H), 6.85 (s, 2H, Ar-H), 6.13 (t, *J* = 6.4 Hz, 1H, C=CH-CH_2_O), 4.60 (d, *J* = 6.0 Hz, 2H, C=CH-CH_2_O), 4.19 (t, *J* = 6.4 Hz, 2H, CH_2_O-Ar), 3.98 (t, *J* = 6.4 Hz, 2H, CH_2_O-N=CH), 3.66 (s, 3H, N-CH_3_), 2.41 (s, 3H, CH_3_), 2.00–2.06 (m, 2H, CH_2_CH_2_CH_2_); ^13^C-NMR (CDCl_3_): δ 156.7, 153.9, 147.1, 146.5, 146.0, 139.6, 139.5, 130.6, 129.8, 125.0, 124.8, 124.6, 120.4, 118.4, 115.2, 112.8, 100.5, 70.5, 70.4, 65.5, 34.3, 29.6, 14.3. Anal. Calcd for C_25_H_22_Cl_4_F_3_N_3_O_4_: C 47.87; H 3.54; N 6.70. Found: C 47.69; H 3.73; N 6.52.

*1-Methyl-3-methyl-5-(2,4-dichlorophenoxy)-1H-pyrazole-4-carbaldehyde-O-{3-[2,6-dichloro-4-(3,3-dichloroallyoxy)-phenoxy]propyl}-oxime* (**7m**)*:* Yellow oil, yield 60%. ^1^H-NMR (CDCl_3_): δ 7.77 (s, 1H, CH=N), 7.48 (d, *J* = 2.4 Hz, 1H, Ar-H), 7.13–7.16 (m, 1H, Ar-H), 6.85 (s, 2H, Ar-H), 6.65 (d, *J* = 8.8 Hz, 1H, Ar-H), 6.14 (t, *J* = 6.4 Hz, 1H, C=CH-CH_2_O), 4.60 (d, *J* = 6.4 Hz, 2H, C=CH-CH_2_O), 4.21 (t, *J* = 6.4 Hz, 2H, CH_2_O-Ar), 4.01 (t, *J* = 6.4 Hz, 2H, CH_2_O-N=CH), 3.67 (s, 3H, N-CH_3_), 2.37 (s, 3H, CH_3_), 2.05–2.09 (m, 2H, CH_2_CH_2_CH_2_); ^13^C-NMR (CDCl_3_): δ 153.9, 151.0, 147.1, 146.5, 146.0, 144.5, 143.7, 139.5, 130.6, 129.8, 127.9, 125.0, 124.6, 123.7, 116.3, 115.2, 100.2, 70.6, 70.5, 65.5, 34.3, 29.6, 14.3. Anal. Calcd for C_24_H_21_Cl_6_N_3_O_4_: C 45.89; H 3.37; N 6.69. Found: C 45.72; H 3.51; N 6.85.

*1-Methyl-3-methyl-5-(2-fluorophenoxy)-1H-pyrazole-4-carbaldehyde-O-{4-[2,6-dichloro-4-(3,3-dichloroallyoxy)-phenoxy]-n-butyl}-oxime* (**7n**)*:* Yellow oil, yield 54%. ^1^H-NMR (CDCl_3_): δ 7.78 (s, 1H, CH=N), 7.04–7.22 (m, 3H, Ar-H), 6.86 (s, 2H, Ar-H), 6.78–6.82 (m, 1H, Ar-H), 6.14 (t, *J* = 6.0 Hz, 1H, C=CH-CH_2_O), 4.61 (d, *J* = 4.0 Hz, 2H, C=CH-CH_2_O), 3.94–4.06 (m, 4H, CH_2_O-Ar and CH_2_O-N=CH), 3.69 (s, 3H, N-CH_3_), 2.39 (s, 3H, CH_3_), 1.73–1.86 (m, 4H, CH_2_CH_2_CH_2_CH_2_); ^13^C-NMR (CDCl_3_): δ 153.9, 153.2, 152.0 (d, *J* = 247 Hz), 147.2, 147.0, 146.0, 144.3, 144.2, 139.8, 129.8, 125.0, 124.6, 124.4 (d, *J* = 7 Hz), 117.1 (d, *J* = 18 Hz), 116.7, 115.2, 100.0, 73.5, 73.4, 65.5, 34.2, 26.5, 25.5, 14.4. Anal. Calcd for C_25_H_24_Cl_4_FN_3_O_4_: C 50.78; H 4.09; N 7.11. Found: C 50.60; H 4.22; N 7.29.

*1-Methyl-3-methyl-5-(4-fluorophenoxy)-1H-pyrazole-4-carbaldehyde-O-{4-[2,6-dichloro-4-(3,3-dichloroallyoxy)-phenoxy]-n-butyl}-oxime* (**7o**): Yellow oil, yield 63%. ^1^H-NMR (CDCl_3_): δ 7.78 (s, 1H, CH=N), 6.87–7.04 (m, 4H, Ar-H), 6.85 (s, 2H, Ar-H), 6.13 (t, *J* = 6.0 Hz, 1H, C=CH-CH_2_O), 4.60 (d, *J* = 6.4 Hz, 2H, C=CH-CH_2_O), 4.07 (t, *J* = 6.0 Hz, 2H, CH_2_O-Ar), 3.95 (t, *J* = 6.0 Hz, 2H, CH_2_O-N=CH), 3.63 (s, 3H, N-CH_3_), 2.39 (s, 3H, CH_3_), 1.79–1.89 (m, 4H, CH_2_CH_2_CH_2_CH_2_); ^13^C-NMR (CDCl_3_): δ 153.9, 146.9, 146.3 (d, *J* = 250 Hz), 146.0, 136.7, 129.8, 125.0, 124.6, 116.7, 116.4, 115.2, 100.5, 73.6, 73.3, 65.5, 34.2, 26.5, 25.6, 14.4. Anal. Calcd for C_25_H_24_Cl_4_FN_3_O_4_: C 50.78; H 4.09; N 7.11. Found: C 50.95; H 3.92; N 7.23.

*1-Methyl-3-methyl-5-(2,4-difluorophenoxy)-1H-pyrazole-4-carbaldehyde-O-{4-[2,6-dichloro-4-(3,3-dichloroallyoxy)-phenoxy]-n-butyl}-oxime* (**7p**)*:* Yellow oil, yield 56%. ^1^H-NMR (CDCl_3_): δ 7.76 (s, 1H, CH=N), 6.96–6.99 (m, 1H, Ar-H), 6.85 (s, 2H, Ar-H), 6.78–6.81 (m, 2H, Ar-H), 6.13 (t, *J* = 6.4 Hz, 1H, C=C**H**-CH_2_O), 4.60 (d, *J* = 6.4 Hz, 2H, C=CH-C**H**_2_O), 4.04 (t, *J* = 6.4 Hz, 2H, CH_2_O-Ar), 3.95 (t, *J* = 6.0 Hz, 2H, C**H**_2_O-N=CH), 3.69 (s, 3H, N-CH_3_), 2.36 (s, 3H, CH_3_), 1.84–1.92 (m, 4H, CH_2_CH_2_CH_2_CH_2_); ^13^C-NMR (CDCl_3_): δ 153.9, 147.1, 147.0, 146.0, 139.4, 129.8, 124.9, 124.6, 117.5, 117.4, 115.2, 111.2, 110.9, 105.8, 105.6, 105.3, 99.8, 73.5, 73.3, 65.4, 34.2, 26.5, 25.5, 14.3. Anal. Calcd for C_25_H_23_Cl_4_F_2_N_3_O_4_: C 49.28; H 3.80; N 6.90. Found: C 49.43; H 3.65; N 6.72.

*1-Methyl-3-methyl-5-(4-fluorophenoxy)-1H-pyrazole-4-carbaldehyde-O-{5-[2,6-dichloro-4-(3,3-dichloroallyoxy)-phenoxy]-n-pentyl}-oxime* (**7q**): Yellow oil, yield 61%. ^1^H-NMR (CDCl_3_z): δ 7.75 (s, 1H, CH=N), 6.98–7.02 (m, 2H, Ar-H), 6.83–6.88 (m, 4H, Ar-H), 6.12 (t, *J* = 6.0 Hz, 1H, C=CH-CH_2_O), 4.58 (d, *J* = 6.0 Hz, 2H, C=CH-CH_2_O), 3.92–4.01 (m, 4H, CH_2_O-Ar and CH_2_O-N=CH), 3.61 (s, 3H, N-CH_3_), 2.37 (s, 3H, CH_3_), 1.80–1.84 (m, 2H, CH_2_), 1.53-1.67 (m, 4H, 2 × CH_2_); ^13^C-NMR (CDCl_3_): δ 149.8, 146.9, 146.1, 139.7, 130.4, 129.8, 125.0, 124.6, 123.0, 119.7, 116.6, 116.5, 116.3, 115.2, 100.3, 73.8, 73.6, 65.5, 34.2, 29.8, 28.8, 22.3, 14.6. Anal. Calcd for C_26_H_26_Cl_4_FN_3_O_4_: C 51.59; H 4.33; N 6.94. Found: C 51.43; H 4.52; N 6.81.

*1-Phenyl-3-methyl-5-(4-fluorophenoxy)-1H-pyrazole-4-carbaldehyde-O-{5-[2,6-dichloro-4-(3,3-dichloroallyoxy)-phenoxy]-n-pentyl}-oxime* (**7r**): Yellow oil, yield 57%. ^1^H-NMR (CDCl_3_): δ 7.82 (s, 1H, CH=N), 7.61 (d, *J* = 8.0 Hz, 2H, Ar-H), 7.27–7.41 (m, 3H, Ar-H), 6.90–7.00 (m, 4H, Ar-H), 6.86 (s, 2H, Ar-H), 6.13 (t, *J* = 6.4 Hz, 1H, C=CH-CH_2_O), 4.60 (d, *J* = 6.4 Hz, 2H, C=CH-CH_2_O), 3.95–4.01 (m, 4H, CH_2_O-Ar and CH_2_O-N=CH), 2.50 (s, 3H, CH_3_), 1.84–1.88 (m, 2H, CH_2_), 1.57–1.72 (m, 4H, 2 × CH_2_); ^13^C-NMR (CDCl_3_): δ 159.9, 157.5, 153.8, 152.6, 148.4, 147.0, 146.1, 139.4, 137.4, 129.8, 129.2, 127.3, 124.6, 122.2, 116.8 (d, *J* = 8 Hz), 116.4 (d, *J* = 24 Hz), 115.2, 102.1, 74.0, 73.5, 65.5, 29.8, 28.8, 22.3, 14.9. Anal. Calcd for C_31_H_28_Cl_4_FN_3_O_4_: C 55.79; H 4.23; N 6.30. Found: C 55.95; H 4.06; N 6.15.

### 3.2. Biological Tests

#### 3.2.1. Bioassay Methods

All bioassays were performed on representative test organisms reared in the laboratory. The bioassay was repeated in triplicate at 25 ± 1 °C. Assessments were made on a dead/alive basis, and mortality rates were corrected using Abbott’s formula. Evaluations were based on a percentage scale of 0–100, where 0 equals no activity and 100 equals total kill. For comparative purposes, the controls Fenpyroximate, Pyridalyl and Imidacloprid were tested under the same conditions. 

#### 3.2.2. Acaricidal Activity against *Tetranychus cinnabarinus*

The acaricidal activities against *Tetranychus cinnabarinus* of the designed compounds were evaluated using the reported procedure [26]. Sieva bean plants with primary leaves expanded to 10 cm were selected and cut back to one plant per pot. A small piece was cut from a leaf taken from the main colony and placed on each leaf of the test plants. This was done about 2 h before treatment to allow the mites to move over to the test plant and to lay eggs. The size of the piece was varied to obtain about 60–100 mites per leaf. At the time of the treatment, the piece of leaf used to transfer the mites was removed and discarded. The mite-infested plant were dipped in the test formulation for 3 s with agitation and set in the hood to dry. Plants were kept for 48 h before the numbers of live and dead adults were counted. The test was run three times and results were averaged. 

#### 3.2.3. Larvicidal Activity against *Oriental Armyworm*

The larvicidal activities of the title compounds against *Oriental armyworm* were evaluated by foliar application [27]. About 2 mg of precisely weighed sample was dissolved in 50 μL DMF, and then diluted with water to get a required solution from 500 μg/mL to 20 μg/mL. Individual corn leaves were placed on moistened pieces of filter paper in Petri dishes. The leaves were then dipped in test solution and allowed to dry. The dishes were infested with 10 third-instar oriental armyworm larvae. Mortality was assessed 48 h after treatment. The test was run three times and results were averaged. 

#### 3.2.4. Insecticidal Activity against *Aphis craccivora*

Insecticidal activities of the target compounds were tested against *Aphis craccivora* by foliar application [28]. About 60 aphids were transferred to the shoot with 3–5 fresh leaves of horsebean. The shoot with aphids was cut and dipped into a required solution from 500 μg/mL to 100 μg/mL of the tested compound for 2 s. After removing extra solutions on the leaf, the aphids were raised in the shoot at 25 °C and 85% relative humidity for 48 h. Each experiment for one compound was triplicated.

## 4. Conclusions

In conclusion, a number of novel pyrazole oxime derivatives containing dichloro-allyloxy-phenol unit were designed and synthesized. Their insecticidal and acaricidal activities were tested. The results indicated that all of the target compounds showed no activities against *Tetranychus cinnabarinus* at a concentration of 500 μg/mL. Interestingly, most of the synthesized compounds displayed good larvicidal activity against *Oriental armyworm* at a concentration of 500 μg/mL. Some of the title compounds possessed good larvicidal activity against *Oriental armyworm* even when the concentration was lowered to 20 μg/mL, particularly, compounds **7f**, **7n**, and **7p** had 100%, 90%, and 90% inhibitory rates against *Oriental armyworm*, respectively, which were comparable to that of the control Pyridalyl. Among these compounds, compound **7q** displayed broad spectrum biological activities, it showed potential insecticidal activity against *Aphis craccivora* besides good larvicidal activity against *Oriental armyworm*. Further structural optimization and biological activities about these pyrazole oximes are well under way.

## Supplementary Materials

Supplementary materials (^1^H-NMR and ^13^C-NMR spectra of pyrazole oxime derivatives **7a**–**7r**) can be accessed at: http://www.mdpi.com/1420-3049/20/12/19811/s1.
