# Supplementary material for: Design, Synthesis and Bioactivities of Novel Dichloro-Allyloxy-Phenol-Containing Pyrazole Oxime Derivatives
Source: Molecules. 2015 Dec 8;20(12):21870–80. doi: 10.3390/molecules201219811 (PMC6332197; doi:10.3390/molecules201219811)
Supplement: Supplementary file 1 [file molecules-20-19811-s001.pdf]

Supplementary Materials: Design, Synthesis and Bioactivities of Novel Dichloro-Allyloxy-Phenol-Containing Pyrazole Oxime Derivatives

Hong Dai, Linyu Ye, Huiyang Zhuang, Baojiang Dai, Yuan Fang and Yujun Shi

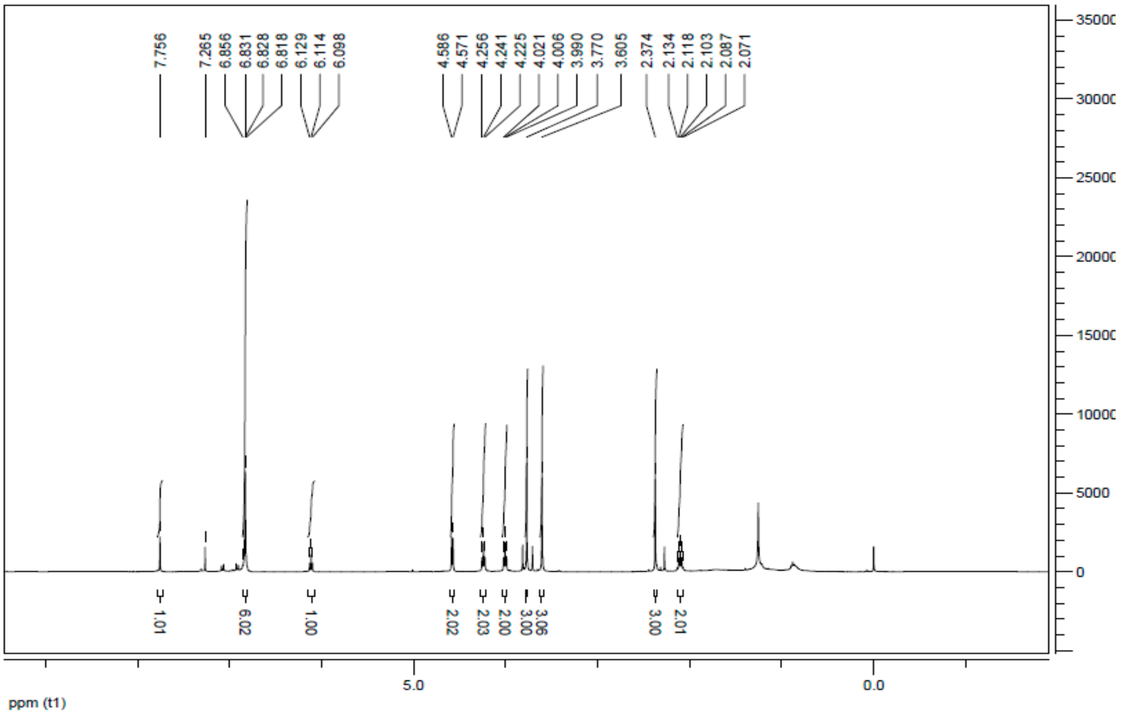

Figure S1. ^{1}H-NMR of compound **7a** (400 MHz, $CDCl_3$).

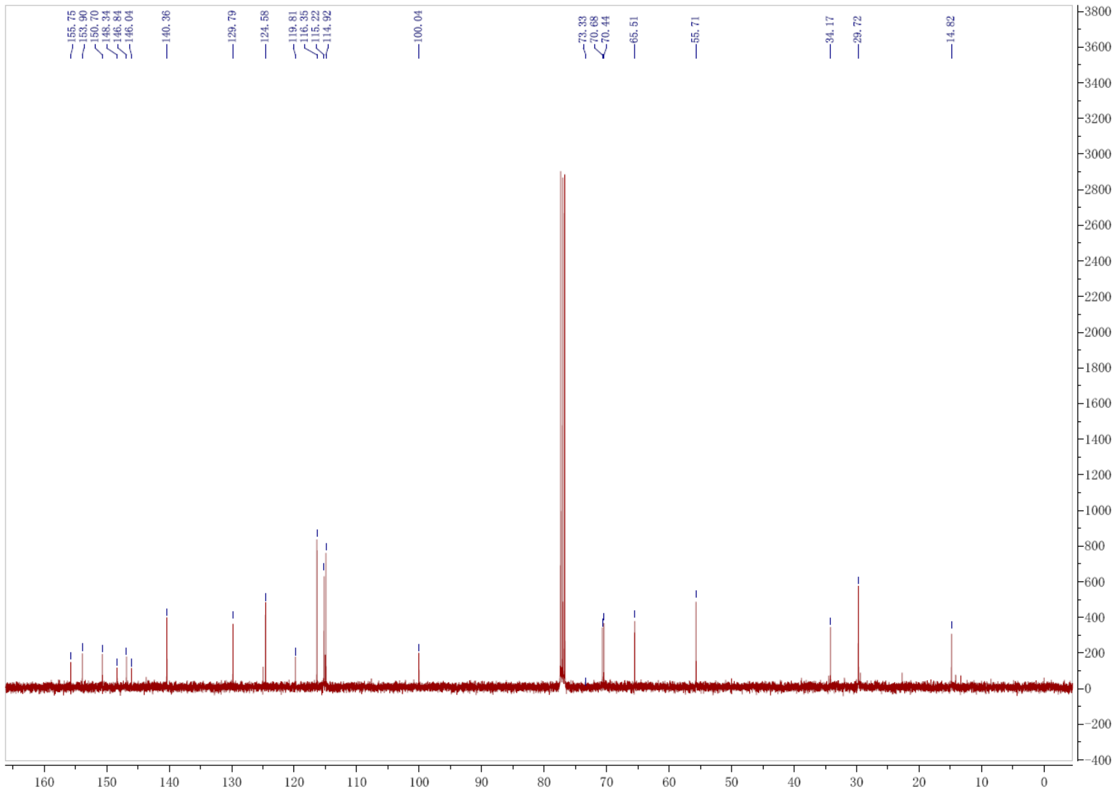

Figure S2. ^{13}C-NMR of compound **7a** (100 MHz, $CDCl_3$).

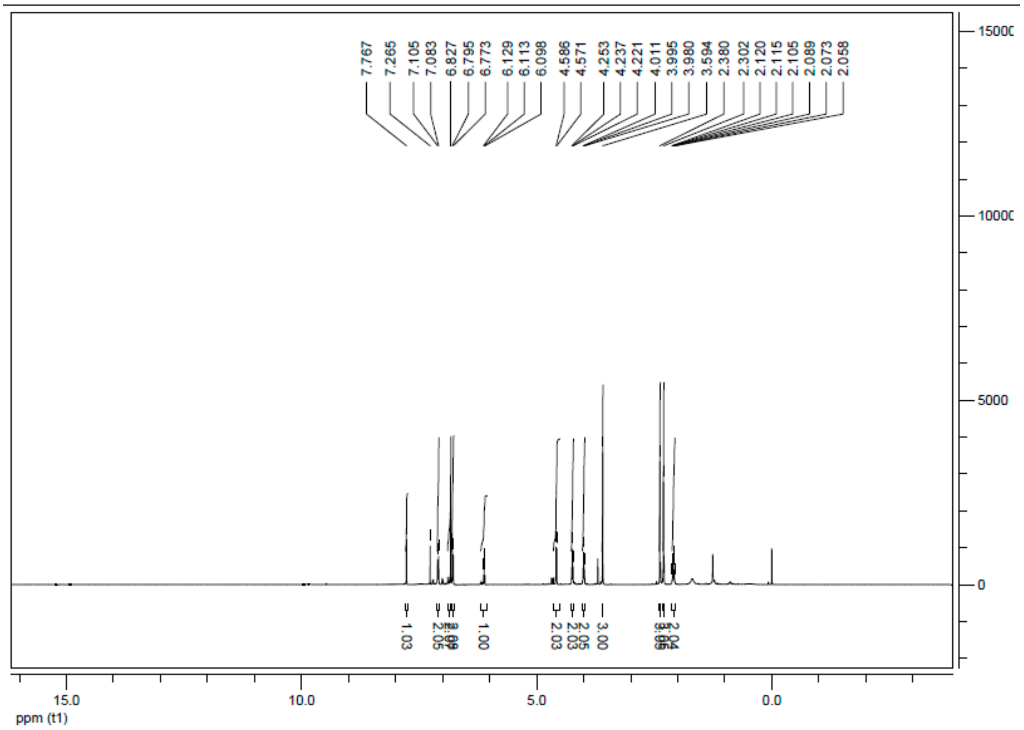

Figure S3. ^{1}H-NMR of compound **7b** (400 MHz, CDCl$_3$).

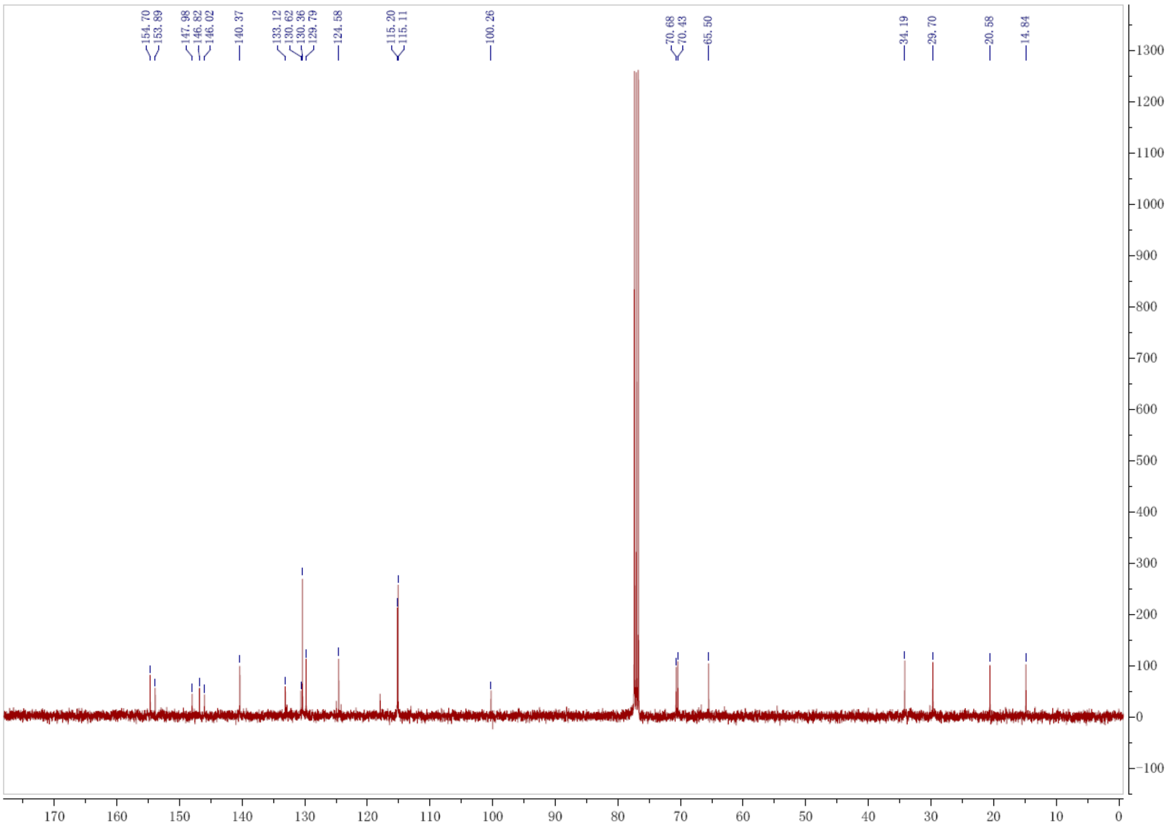

Figure S4. ^{13}C-NMR of compound **7b** (100 MHz, CDCl$_3$).

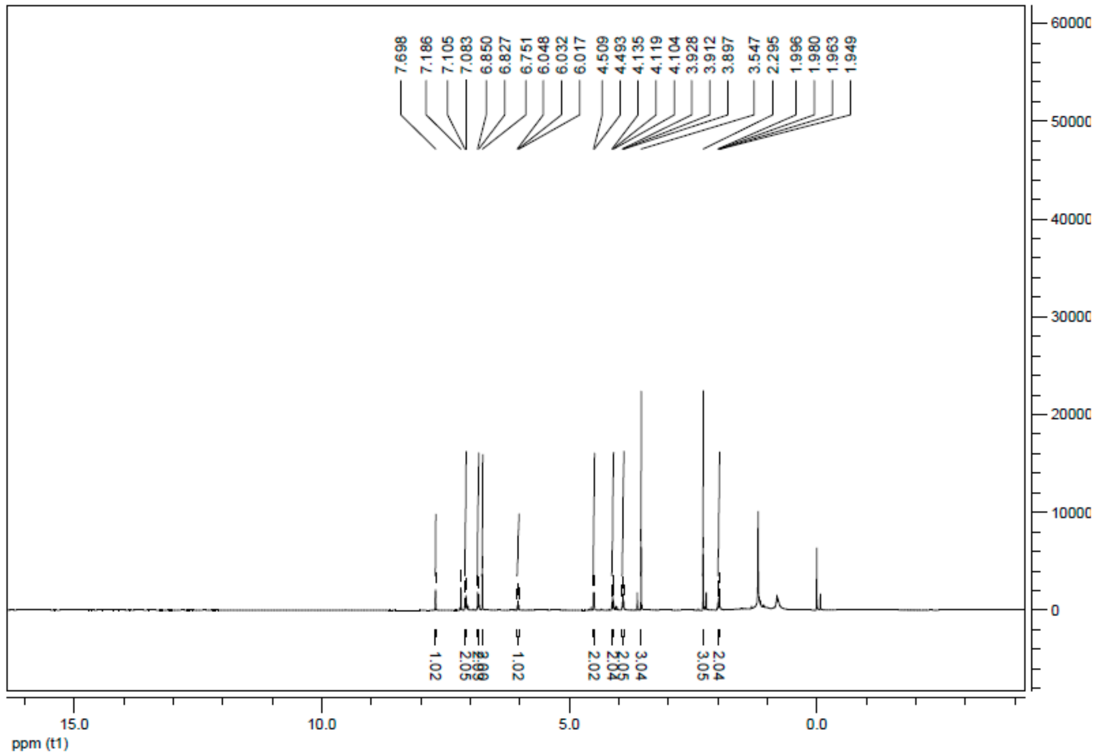

Figure S5. ^{1}H-NMR of compound **7c** (400 MHz, $CDCl_3$).

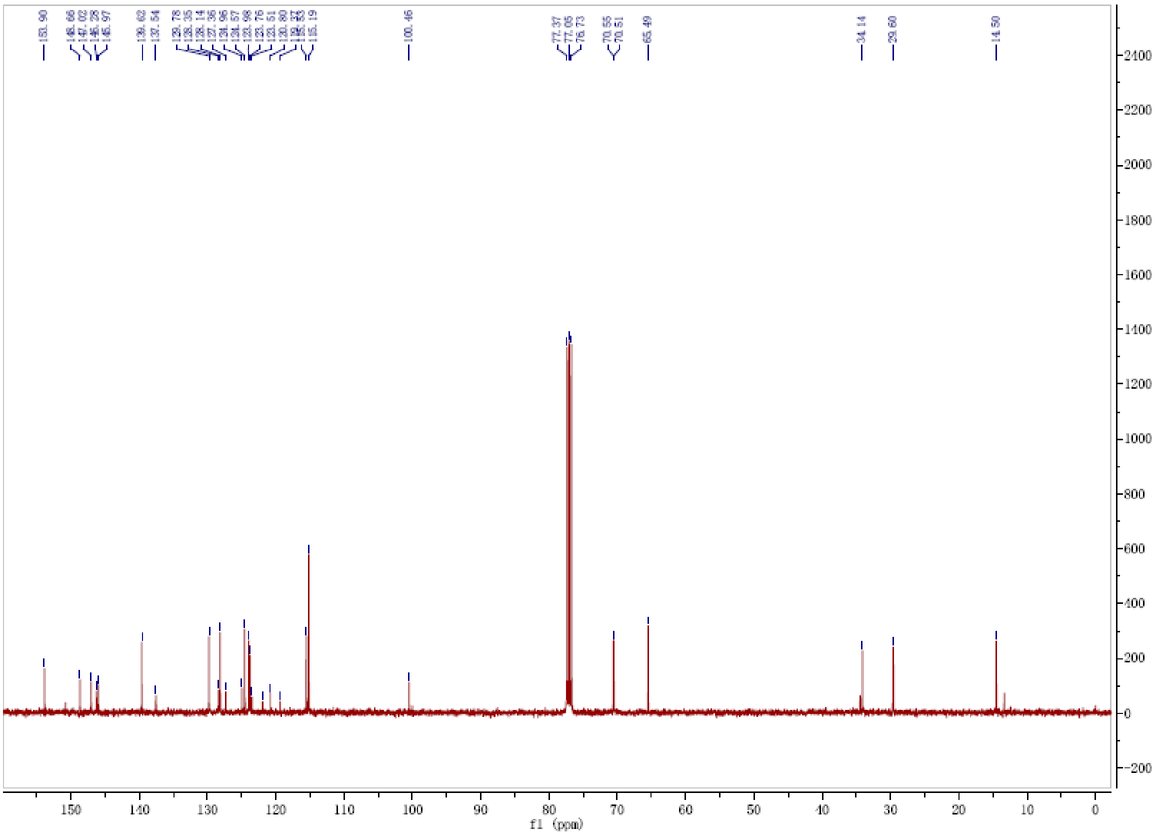

Figure S6. ^{13}C-NMR of compound **7c** (100 MHz, $CDCl_3$).

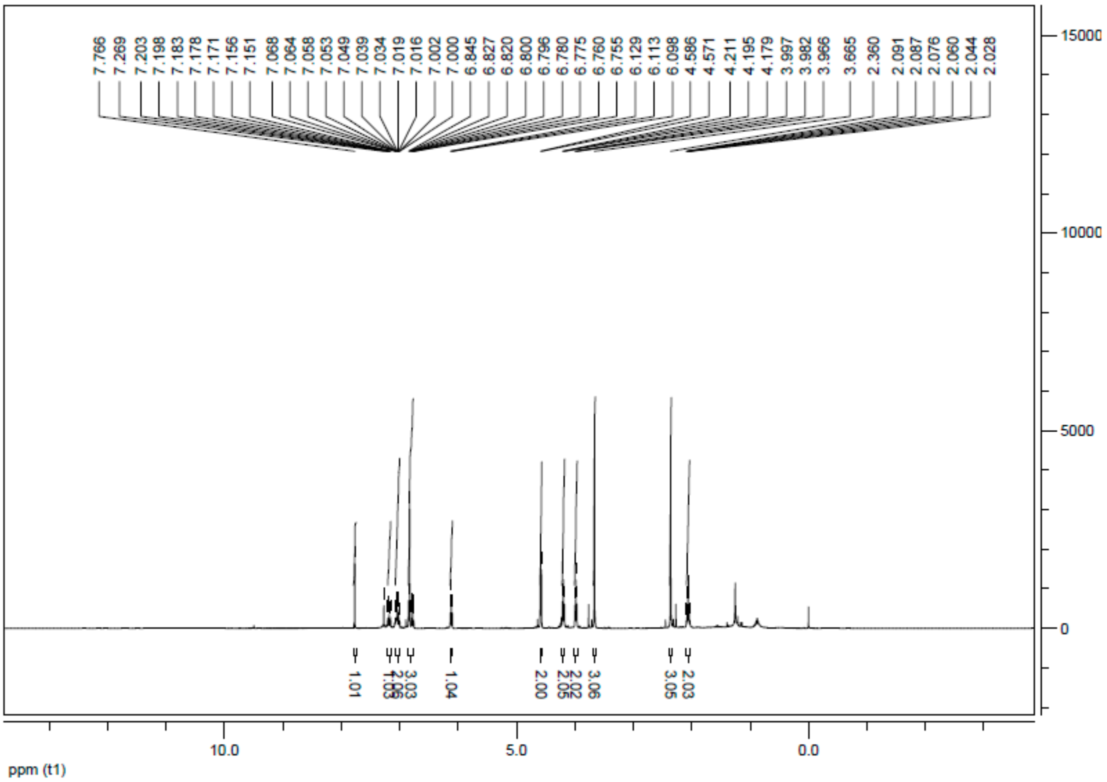

Figure S7. ^{1}H-NMR of compound **7d** (400 MHz, $CDCl_3$).

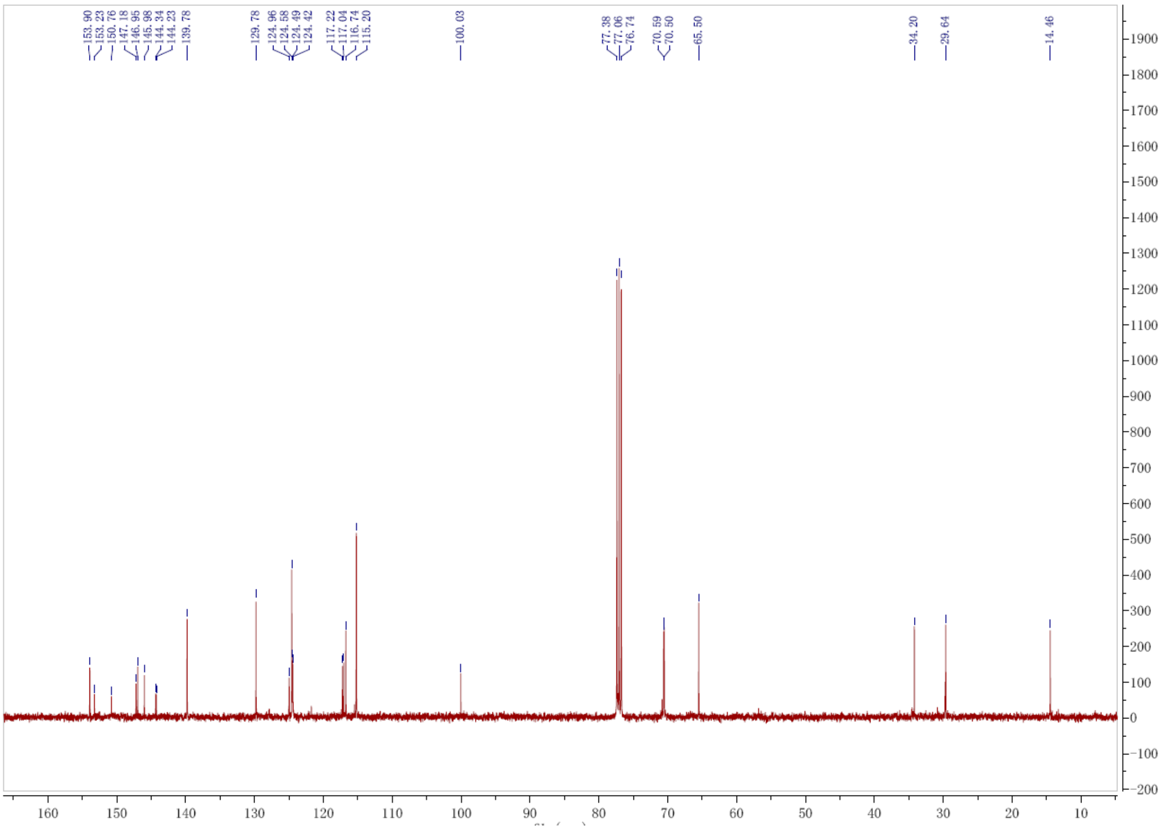

Figure S8. ^{13}C-NMR of compound **7d** (100 MHz, $CDCl_3$).

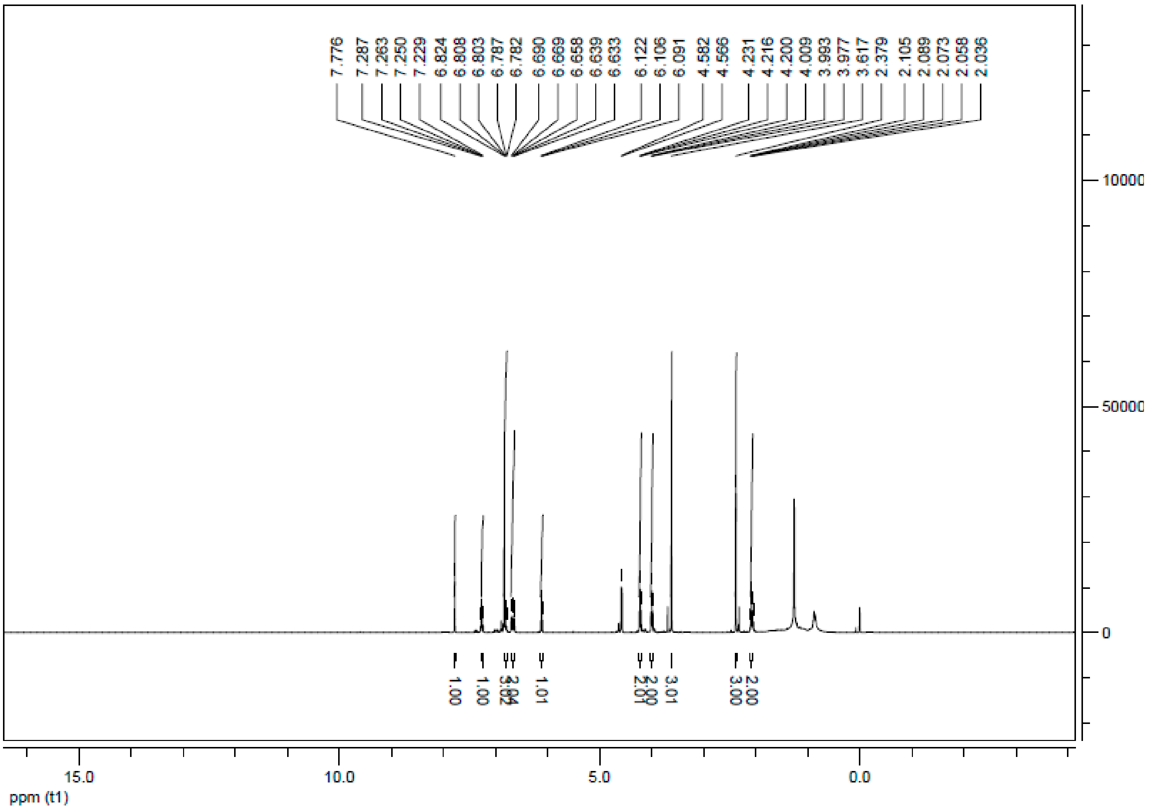

Figure S9. ^{1}H-NMR of compound **7e** (400 MHz, $CDCl_3$).

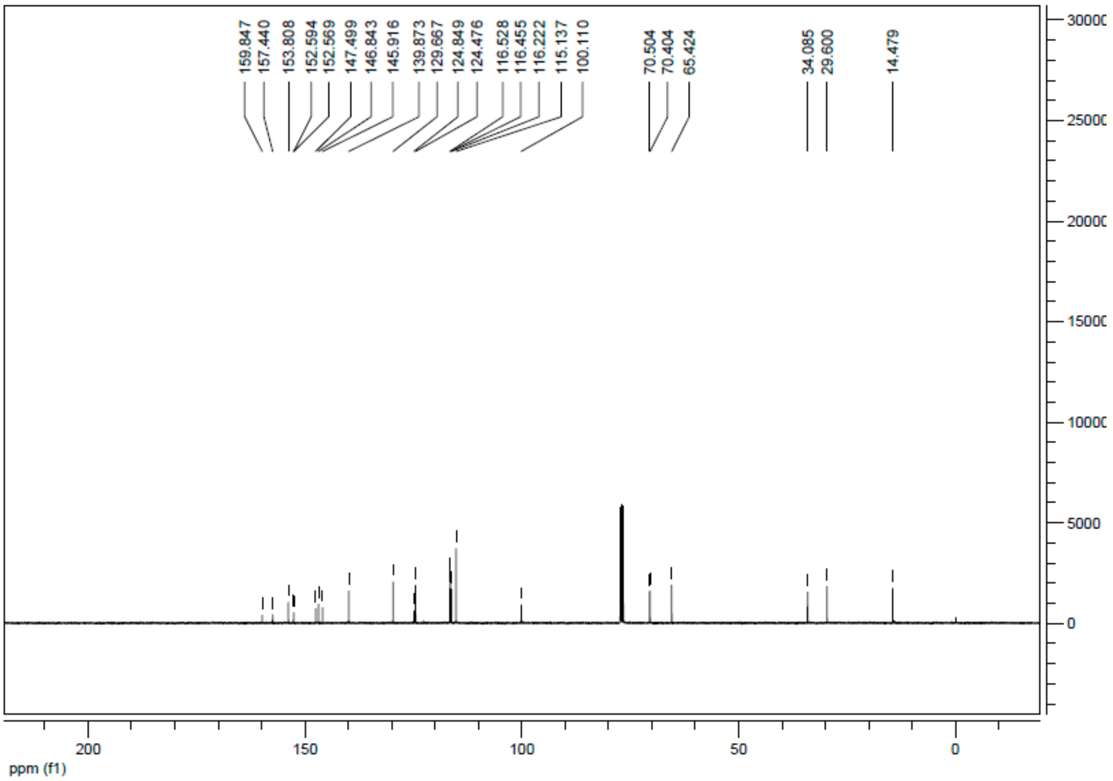

Figure S10. ^{13}C-NMR of compound **7e** (100 MHz, $CDCl_3$).

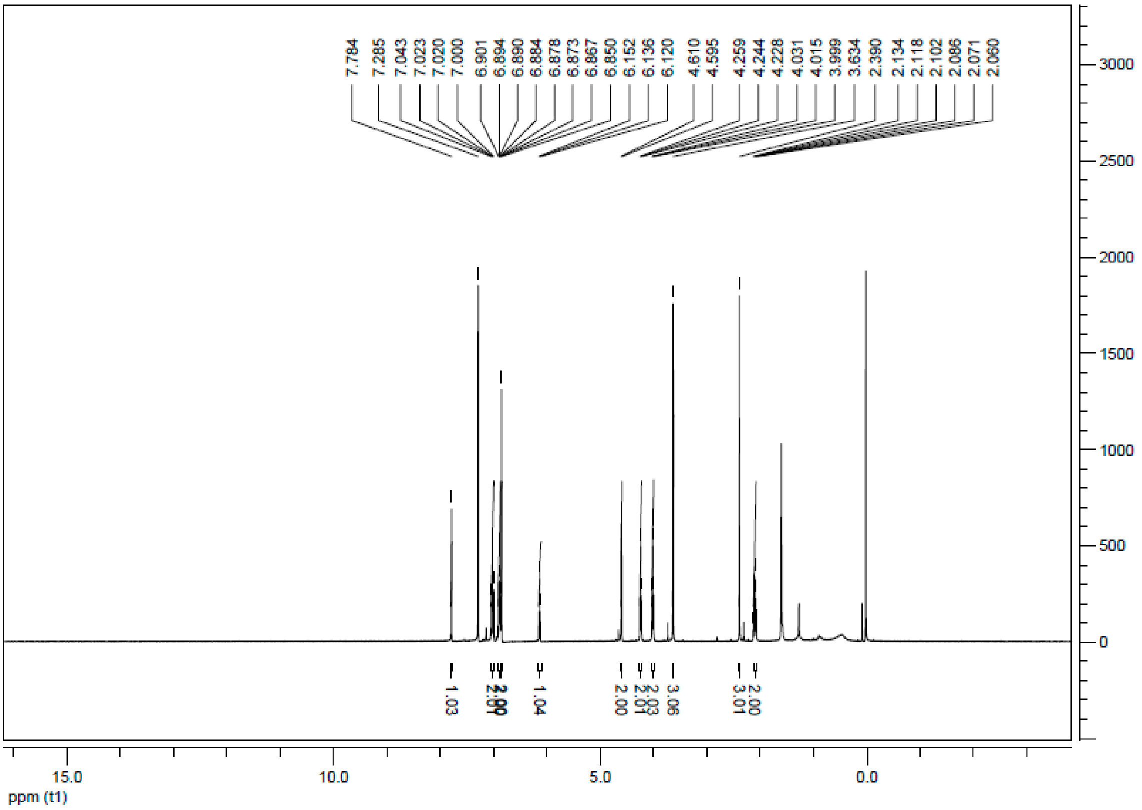

Figure S11. ^{1}H-NMR of compound **7f** (400 MHz, $CDCl_3$).

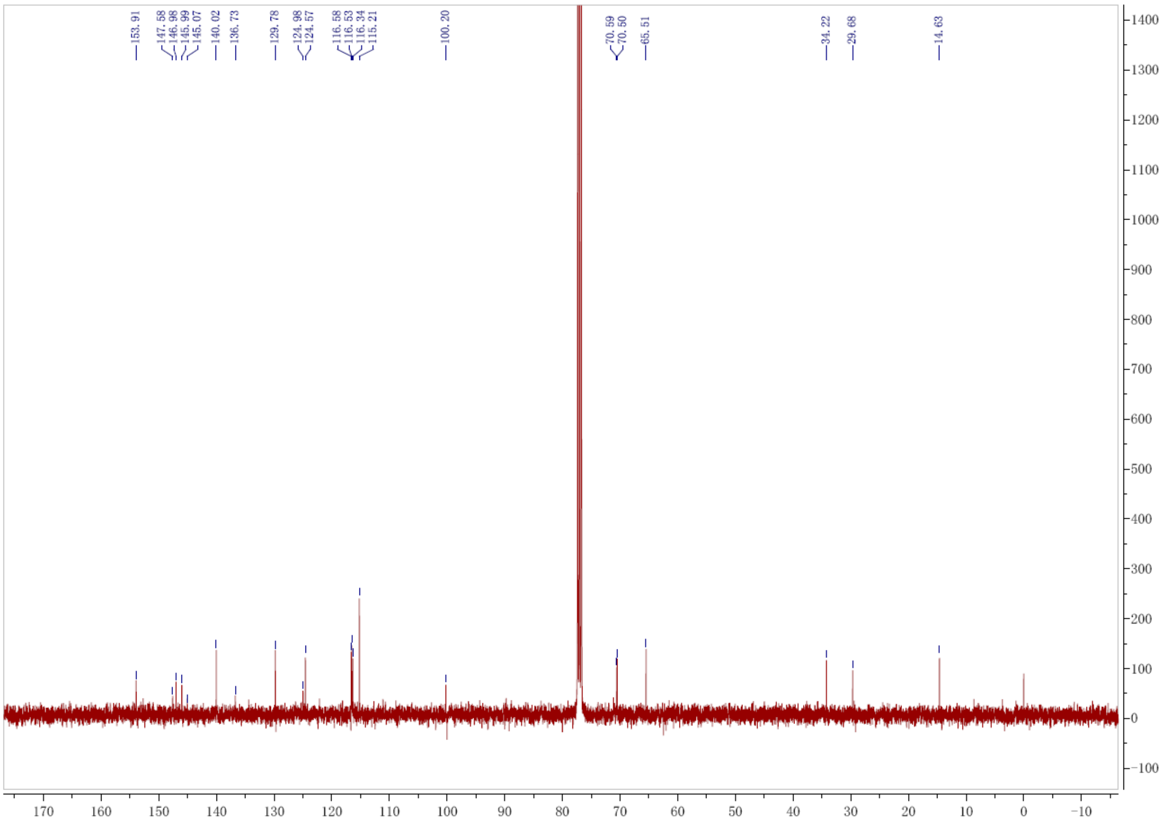

Figure S12. ^{13}C-NMR of compound **7f** (100 MHz, $CDCl_3$).

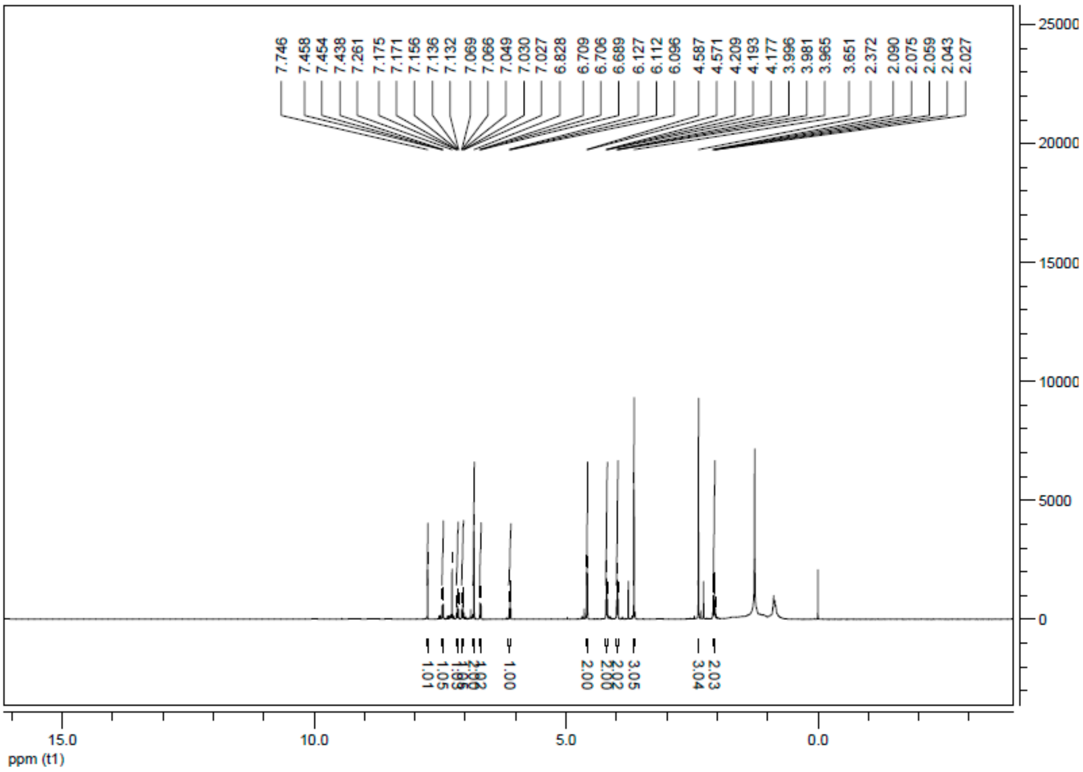

Figure S13. ^{1}H-NMR of compound **7g** (400 MHz, $CDCl_3$).

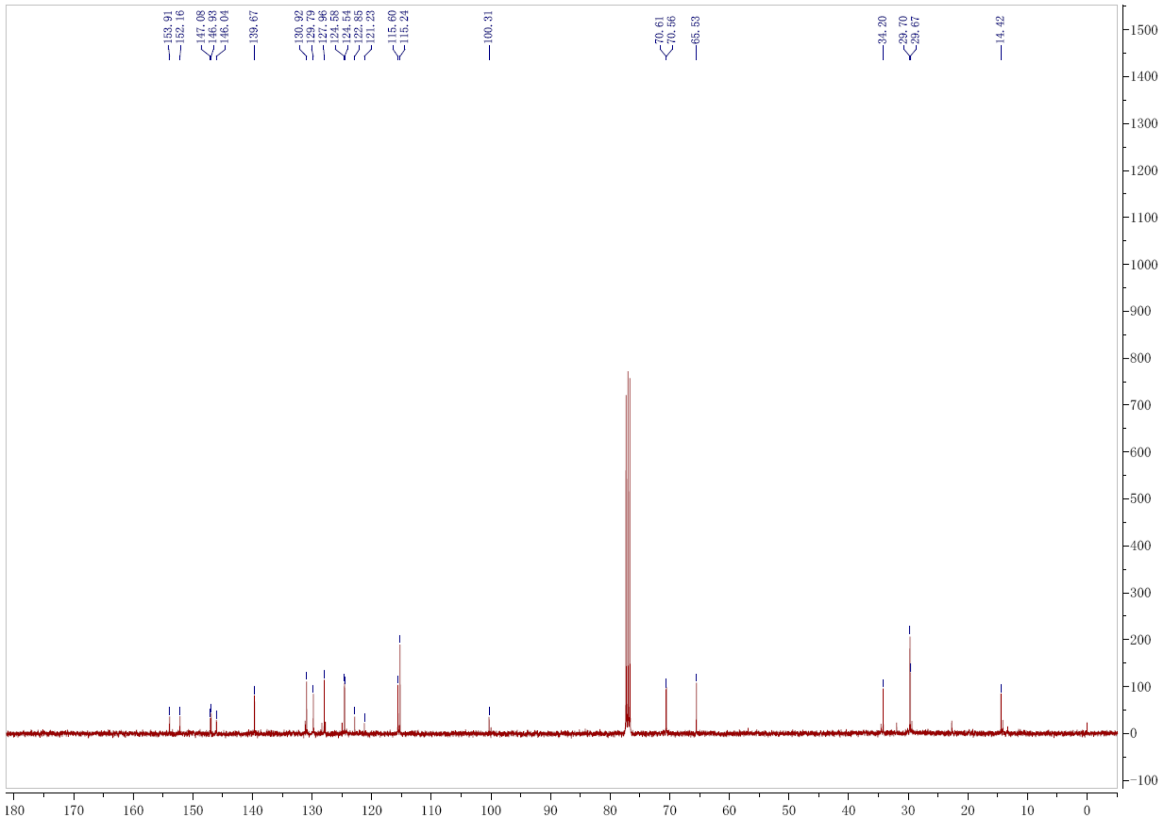

Figure S14. ^{13}C-NMR of compound **7g** (100 MHz, $CDCl_3$).

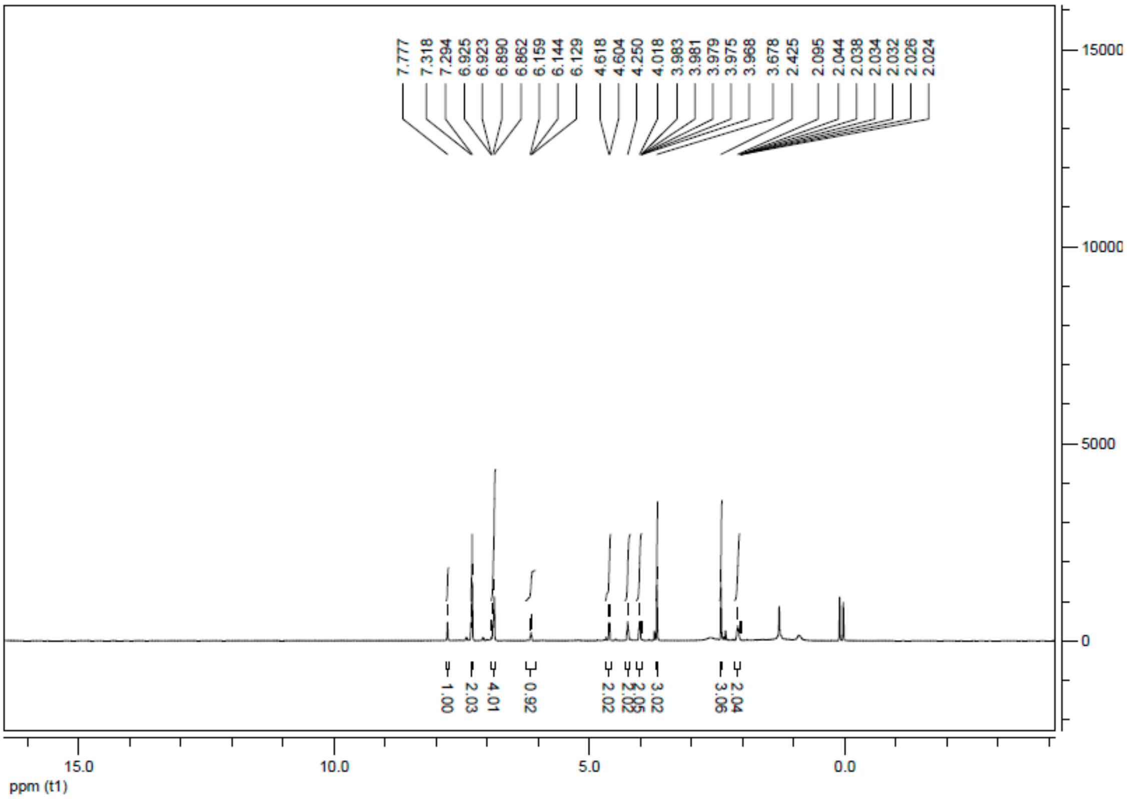

Figure S15. ^{1}H-NMR of compound **7h** (400 MHz, $CDCl_3$).

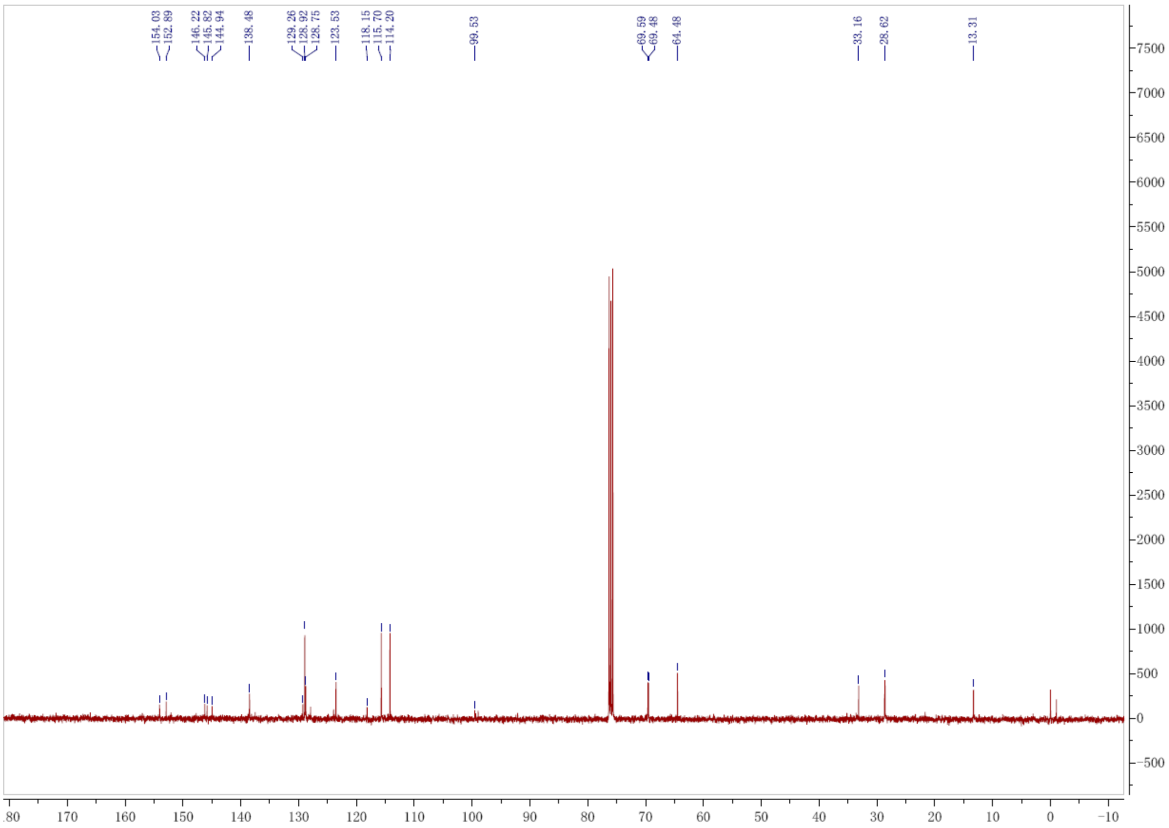

Figure S16. ^{13}C-NMR of compound **7h** (100 MHz, $CDCl_3$).

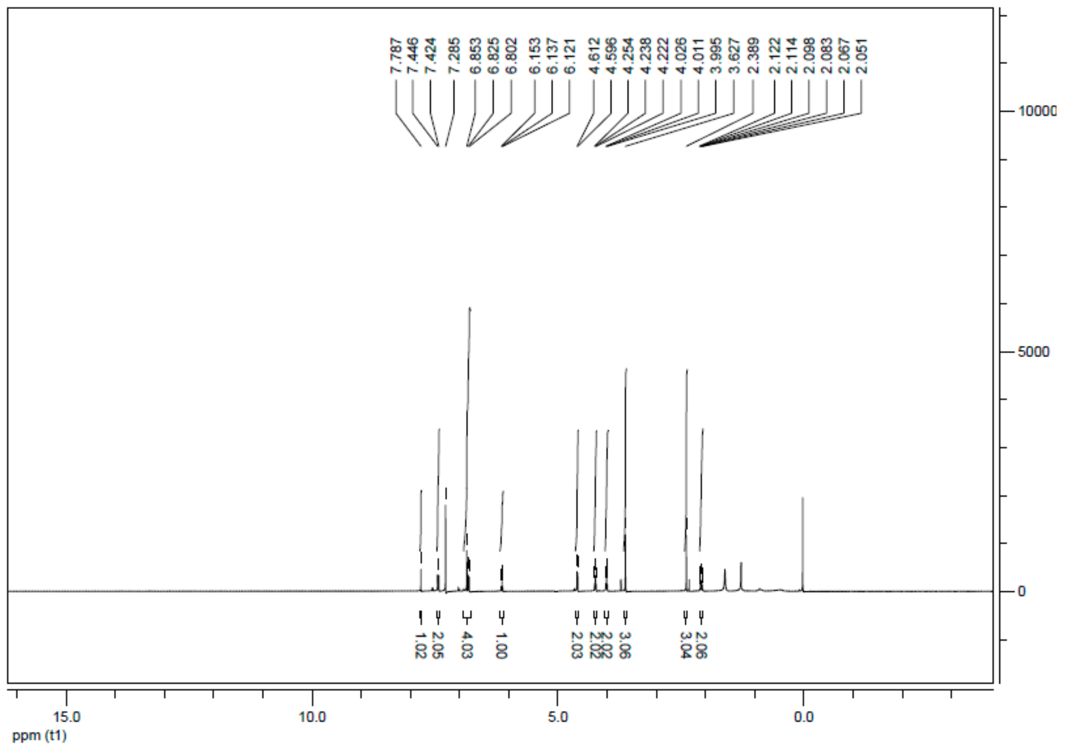

Figure S17. ^{1}H-NMR of compound **7i** (400 MHz, $CDCl_3$).

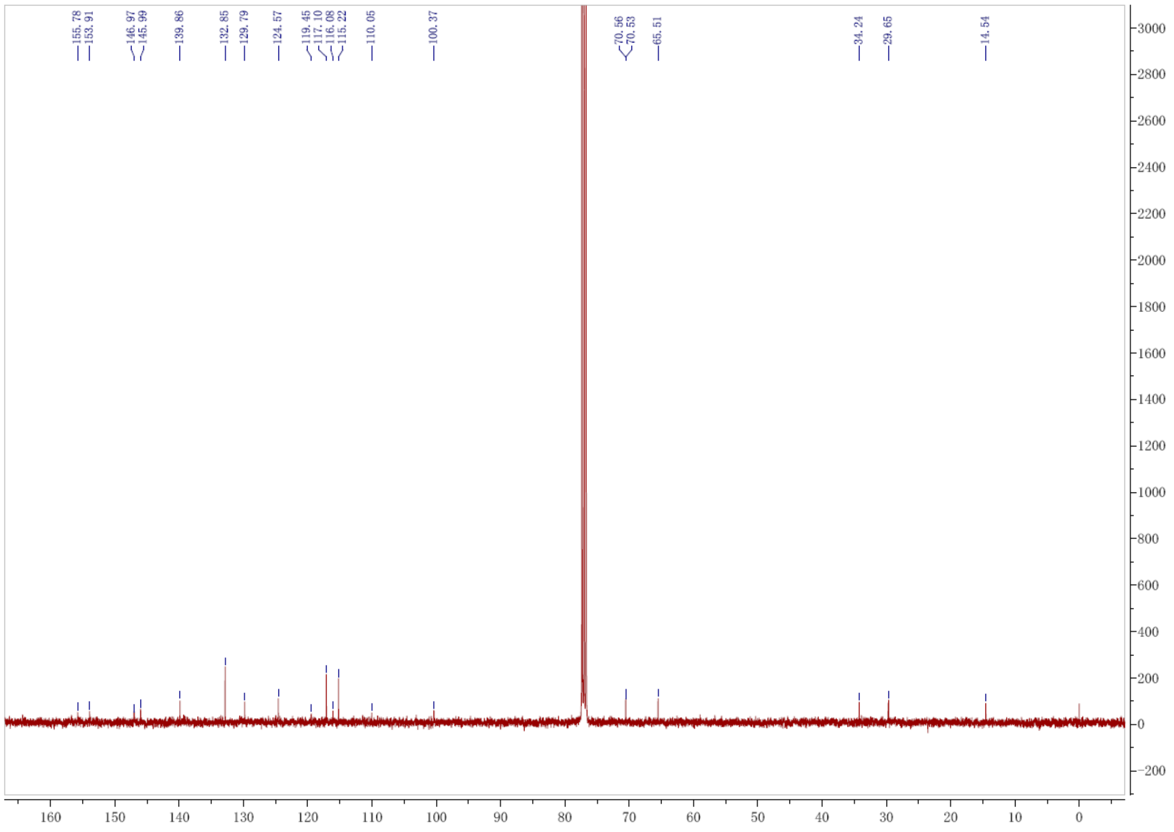

Figure S18. ^{13}C-NMR of compound **7i** (100 MHz, $CDCl_3$).

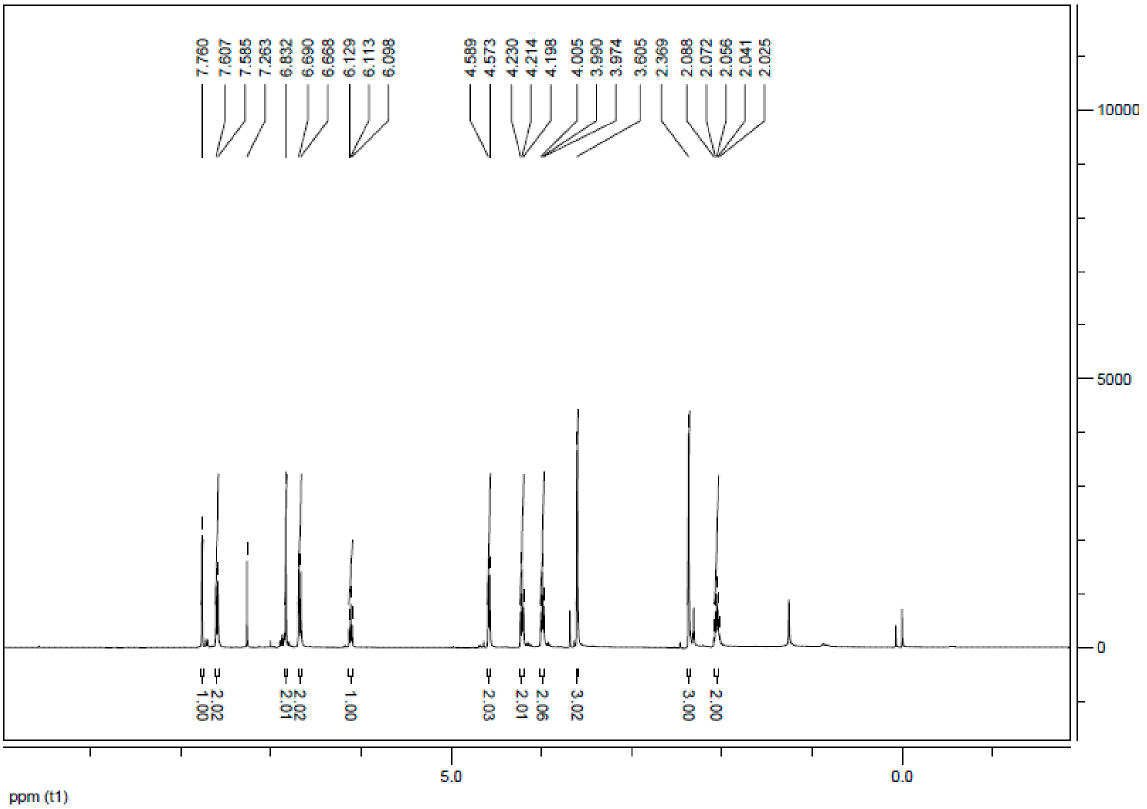

Figure S19. ^{1}H-NMR of compound **7j** (400 MHz, $CDCl_3$).

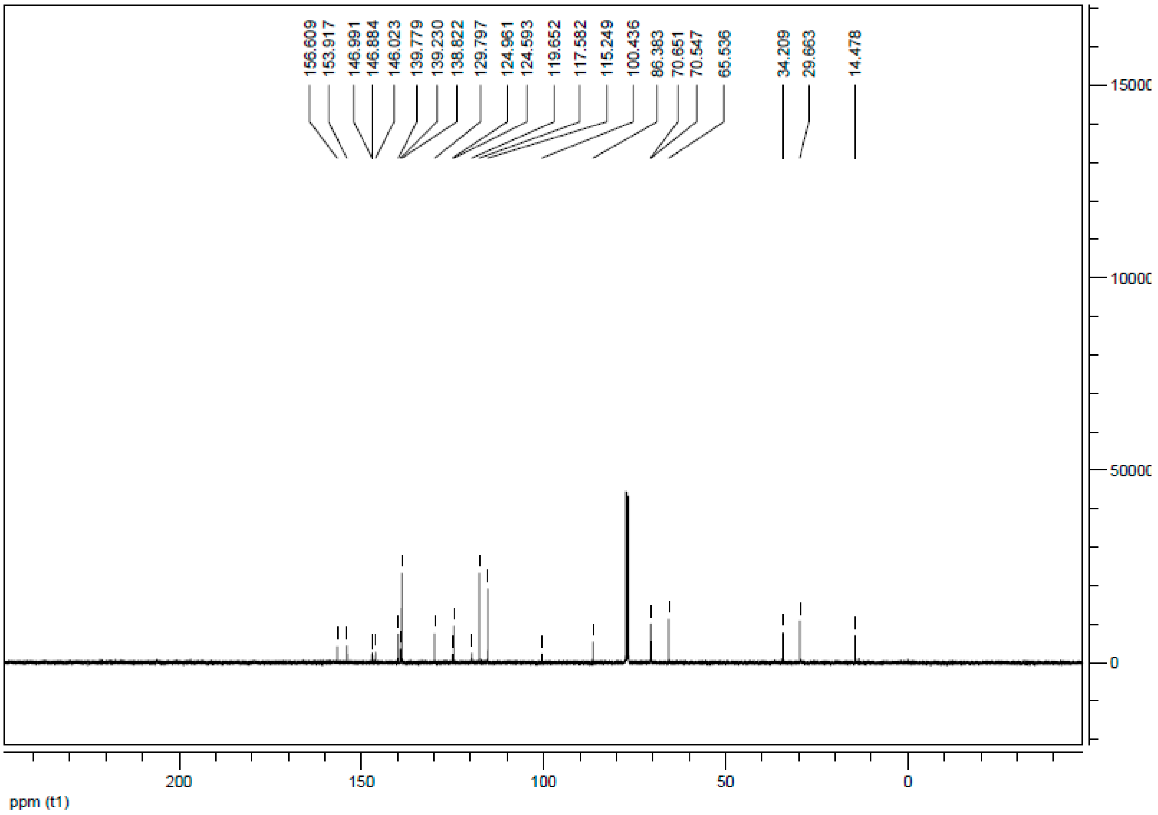

Figure S20. ^{13}C-NMR of compound **7j** (100 MHz, $CDCl_3$).

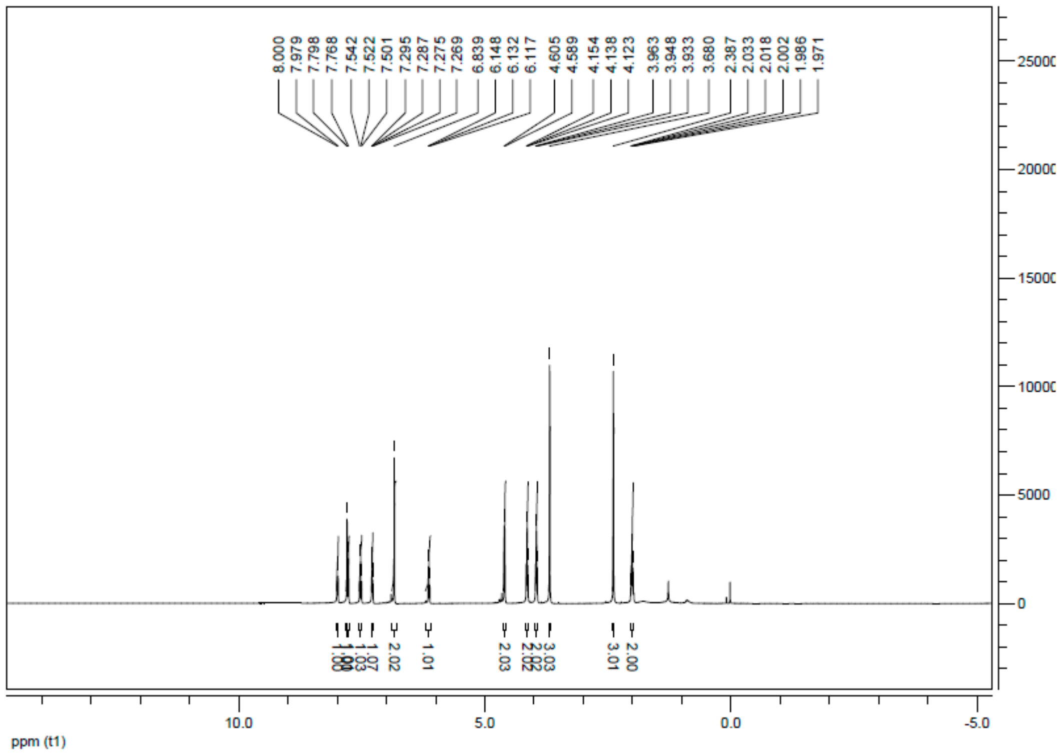

Figure S21. ^{1}H-NMR of compound **7k** (400 MHz, $CDCl_3$).

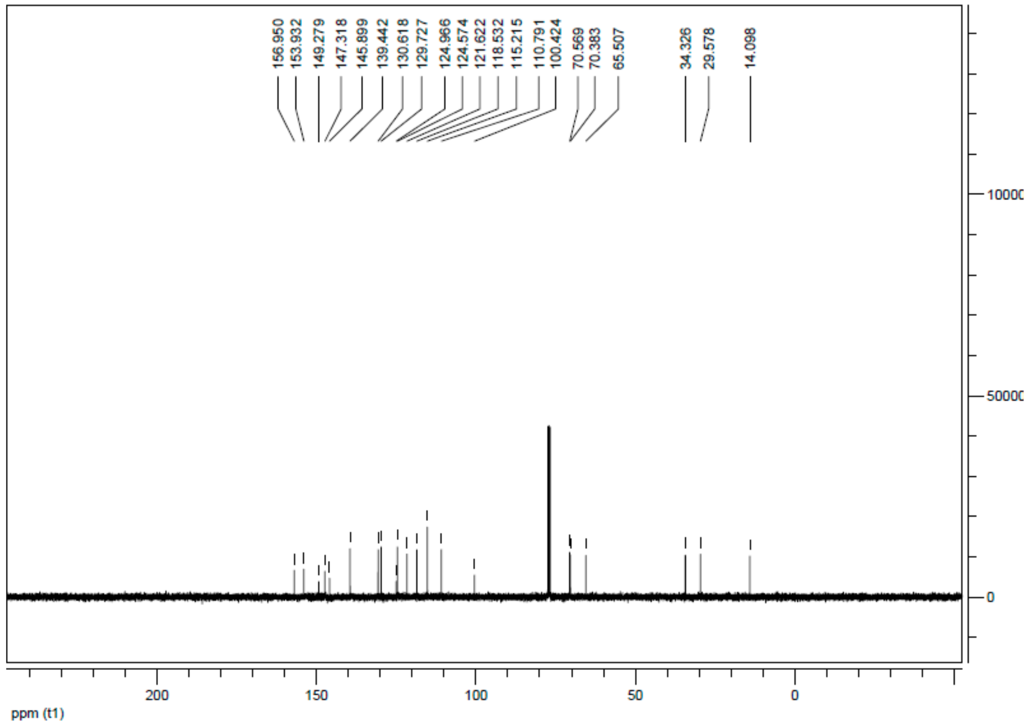

Figure S22. ^{13}C-NMR of compound **7k** (100 MHz, $CDCl_3$).

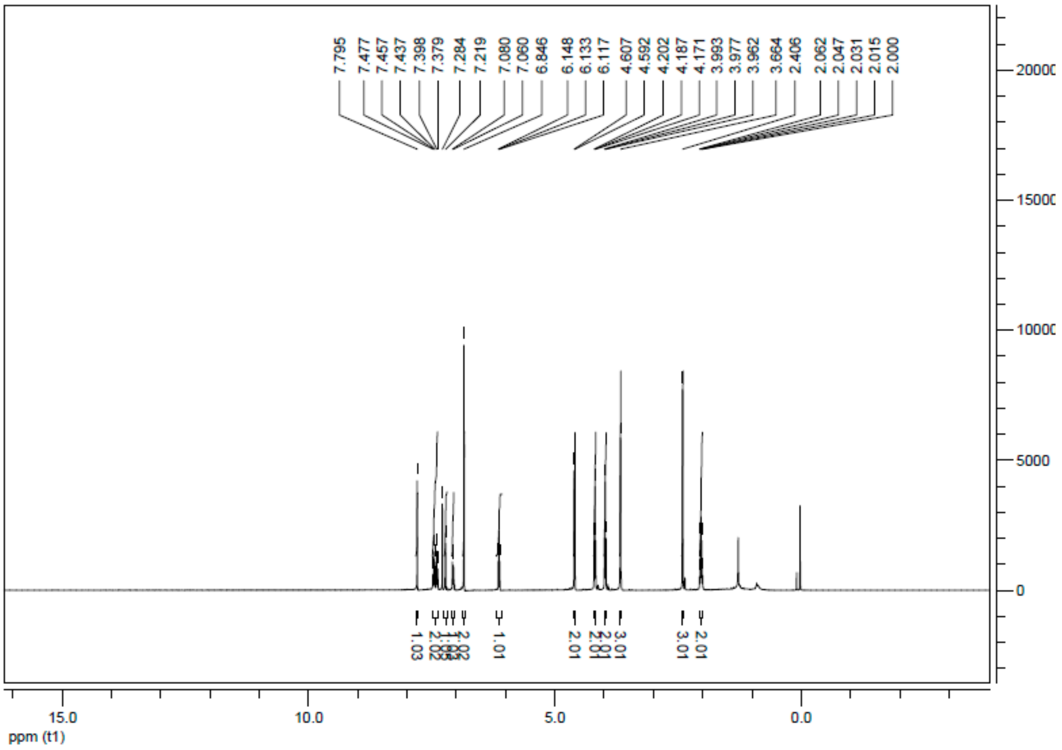

Figure S23. ^{1}H-NMR of compound **7l** (400 MHz, $CDCl_3$).

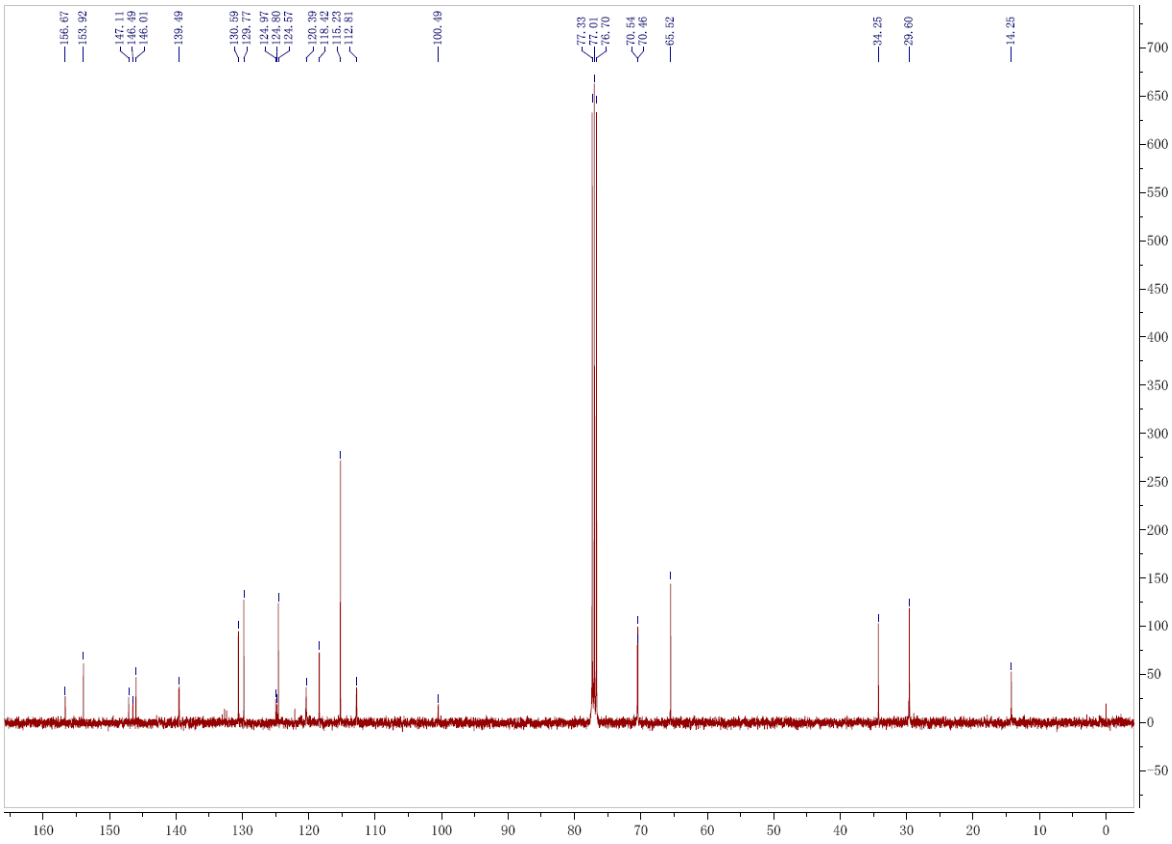

Figure S24. ^{13}C-NMR of compound **7l** (100 MHz, $CDCl_3$).

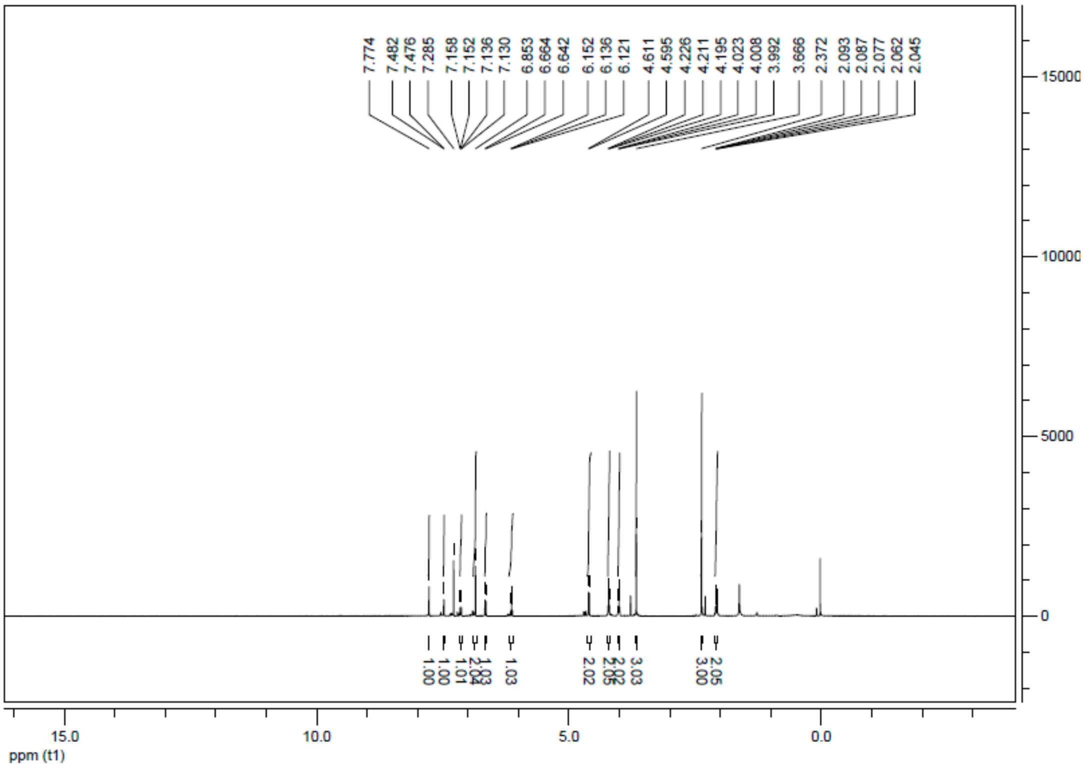

Figure S25. ^{1}H-NMR of compound **7m** (400 MHz, $CDCl_3$).

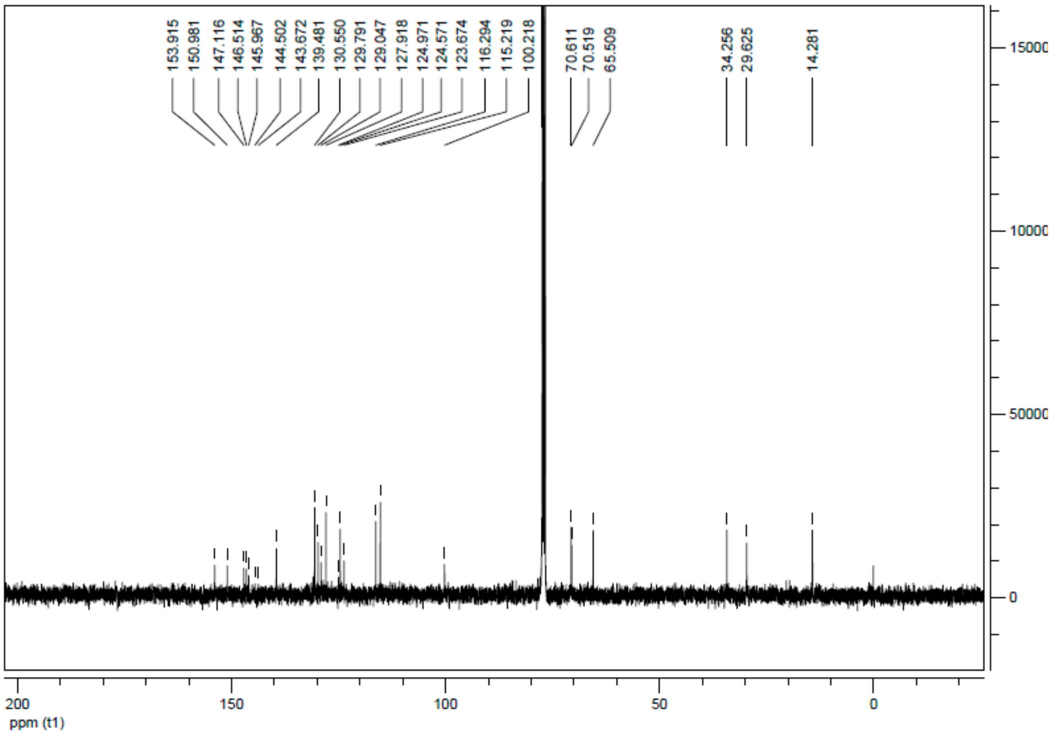

Figure S26. ^{13}C-NMR of compound **7m** (100 MHz, $CDCl_3$).

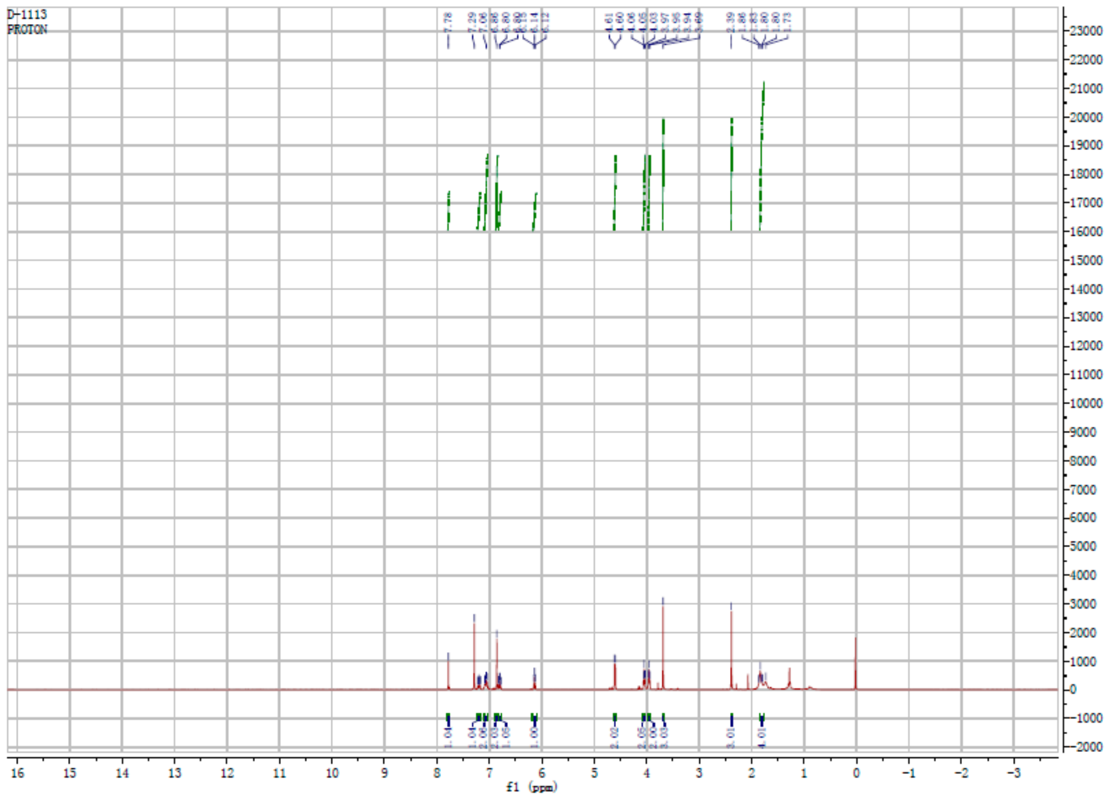

Figure S27. ^{1}H-NMR of compound **7n** (400 MHz, $CDCl_3$).

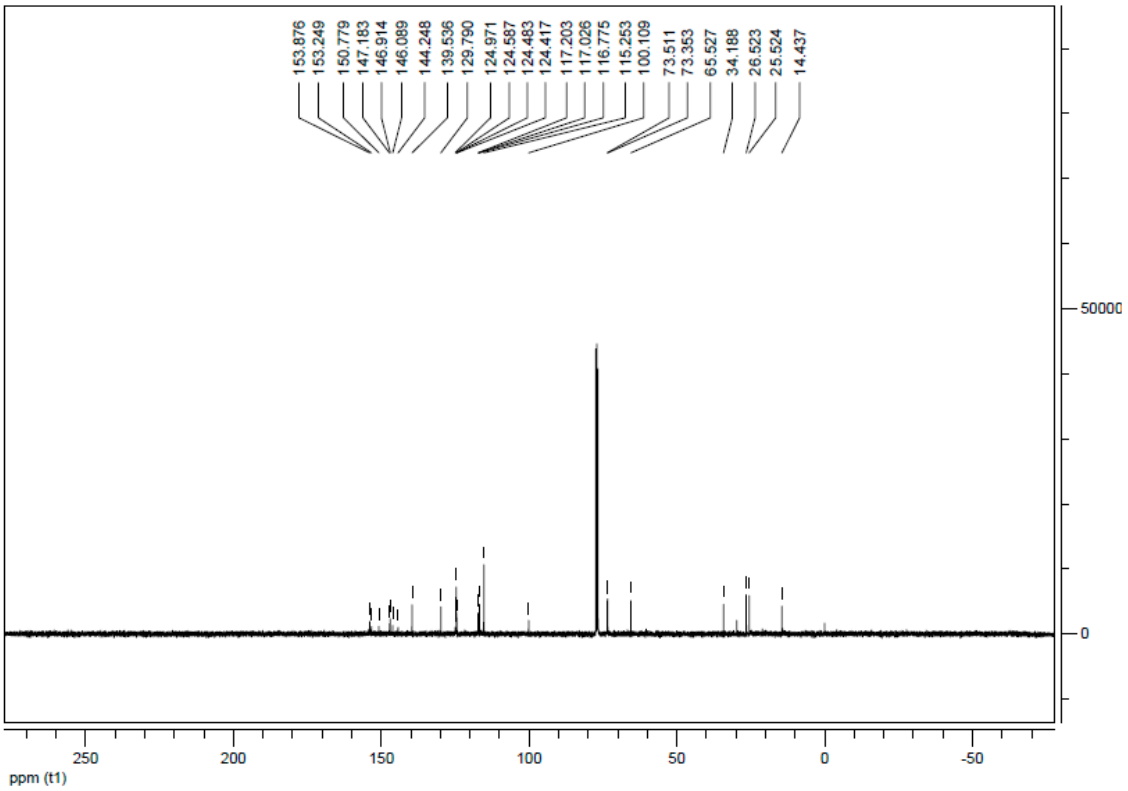

Figure S28. ^{13}C-NMR of compound **7n** (100 MHz, $CDCl_3$).

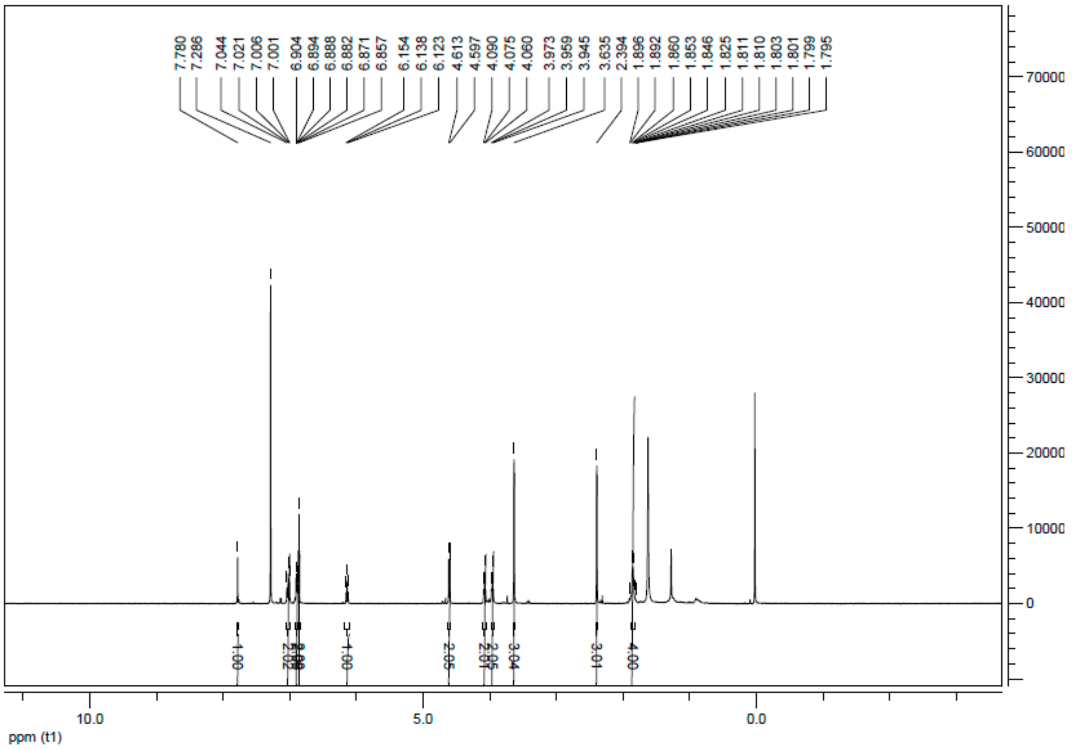

Figure S29. ^{1}H-NMR of compound **7o** (400 MHz, $CDCl_3$).

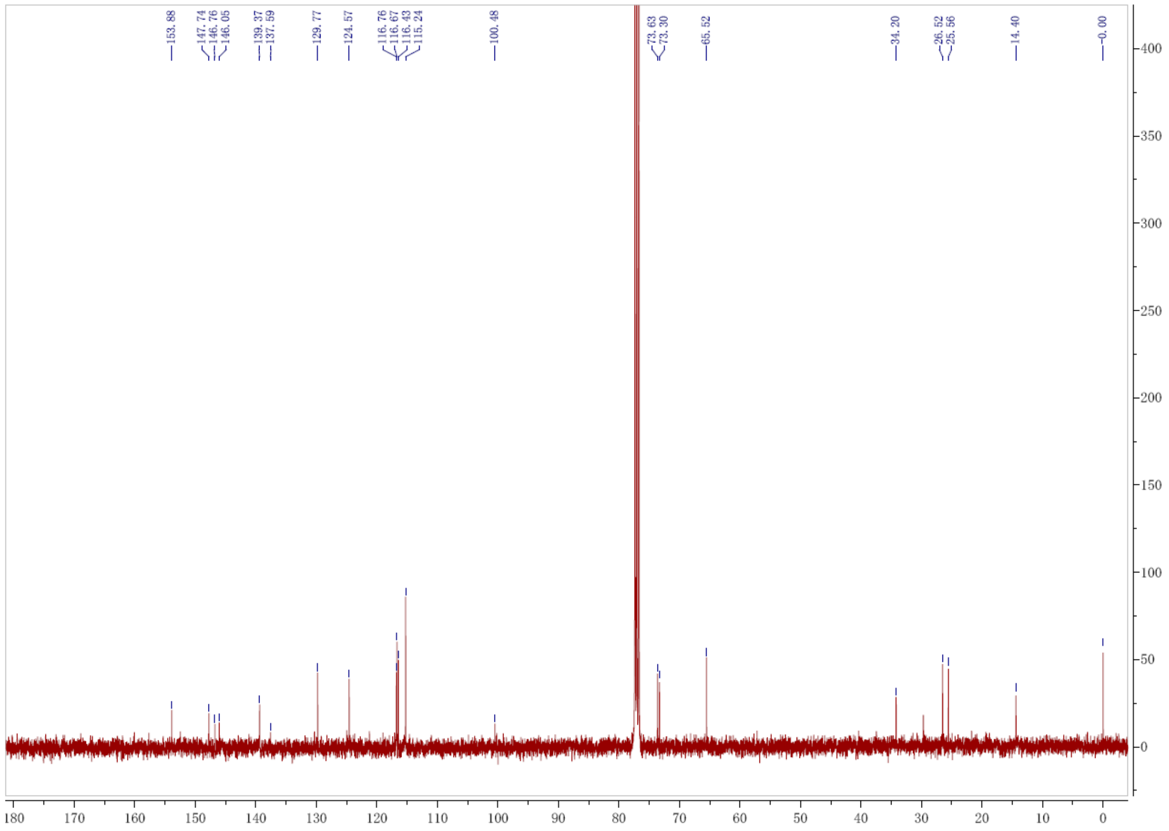

Figure S30. ^{13}C-NMR of compound **7o** (100 MHz, $CDCl_3$).

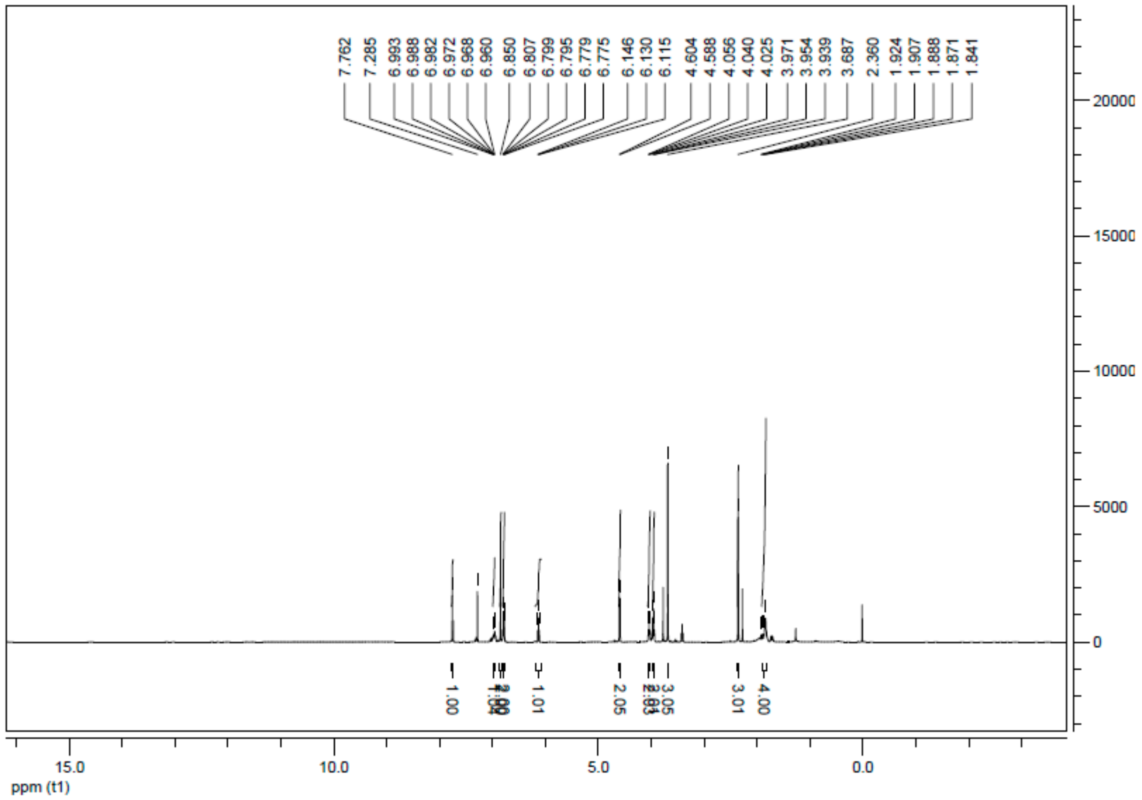

Figure S31. ^{1}H-NMR of compound **7p** (400 MHz, $CDCl_3$).

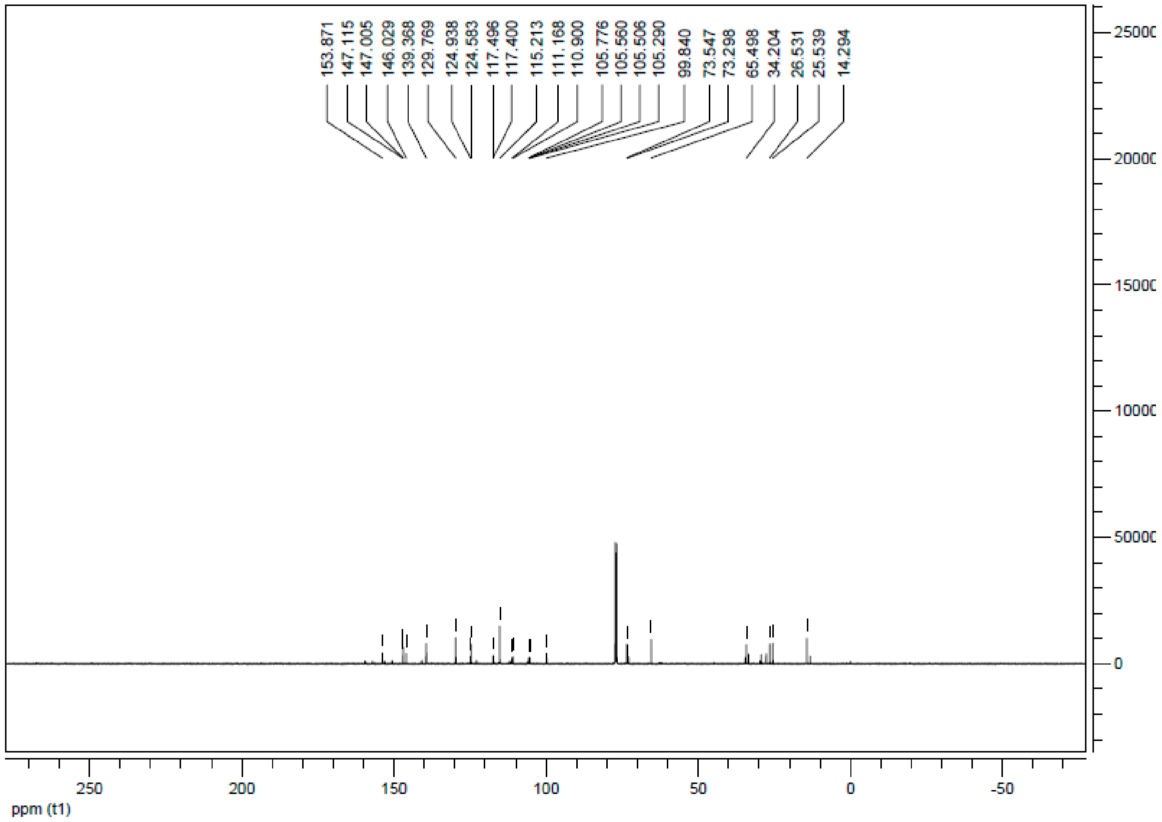

Figure S32. ^{13}C-NMR of compound **7p** (100 MHz, $CDCl_3$).

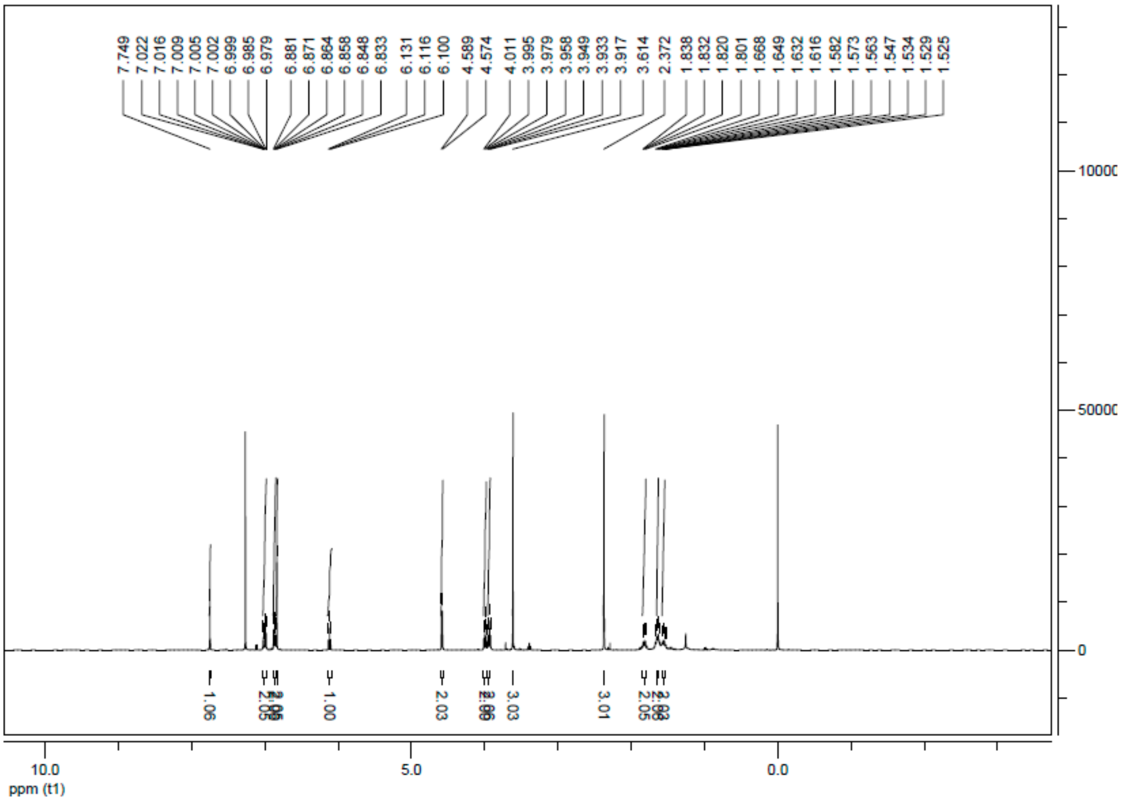

Figure S33. ^{1}H-NMR of compound **7q** (400 MHz, $CDCl_3$).

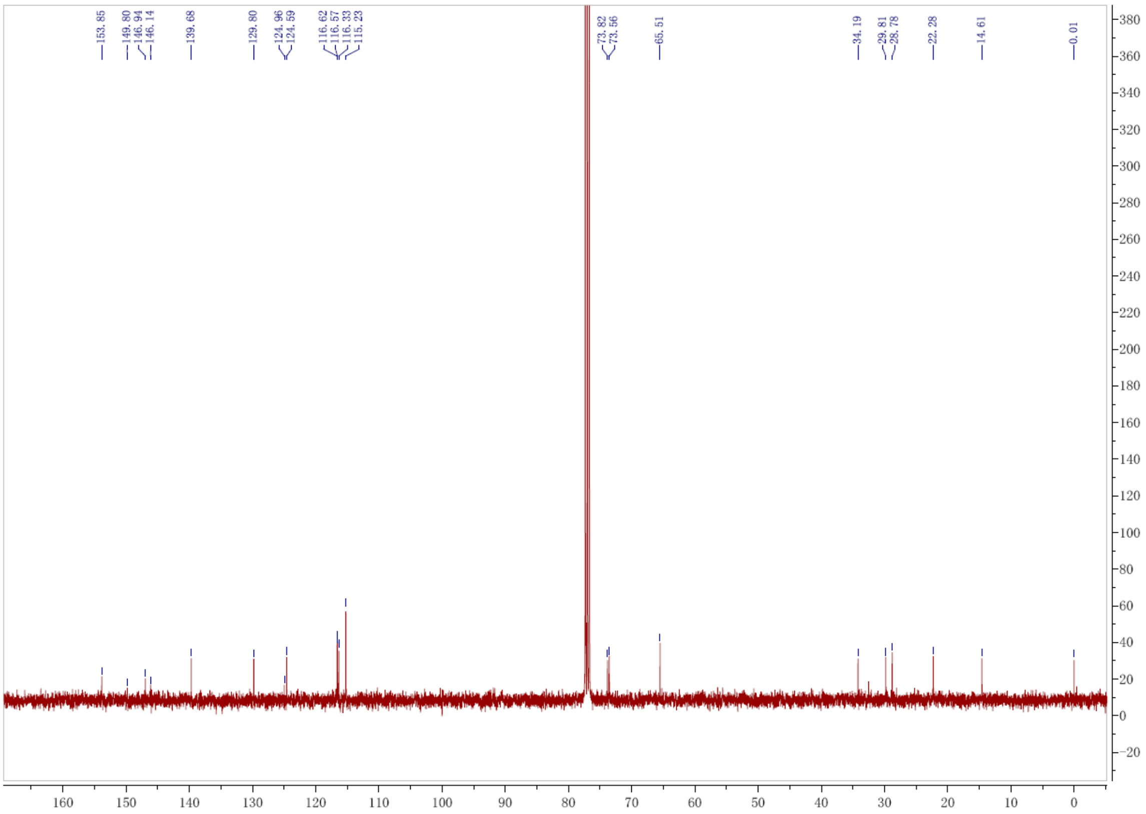

Figure S34. ^{13}C-NMR of compound **7q** (100 MHz, $CDCl_3$).

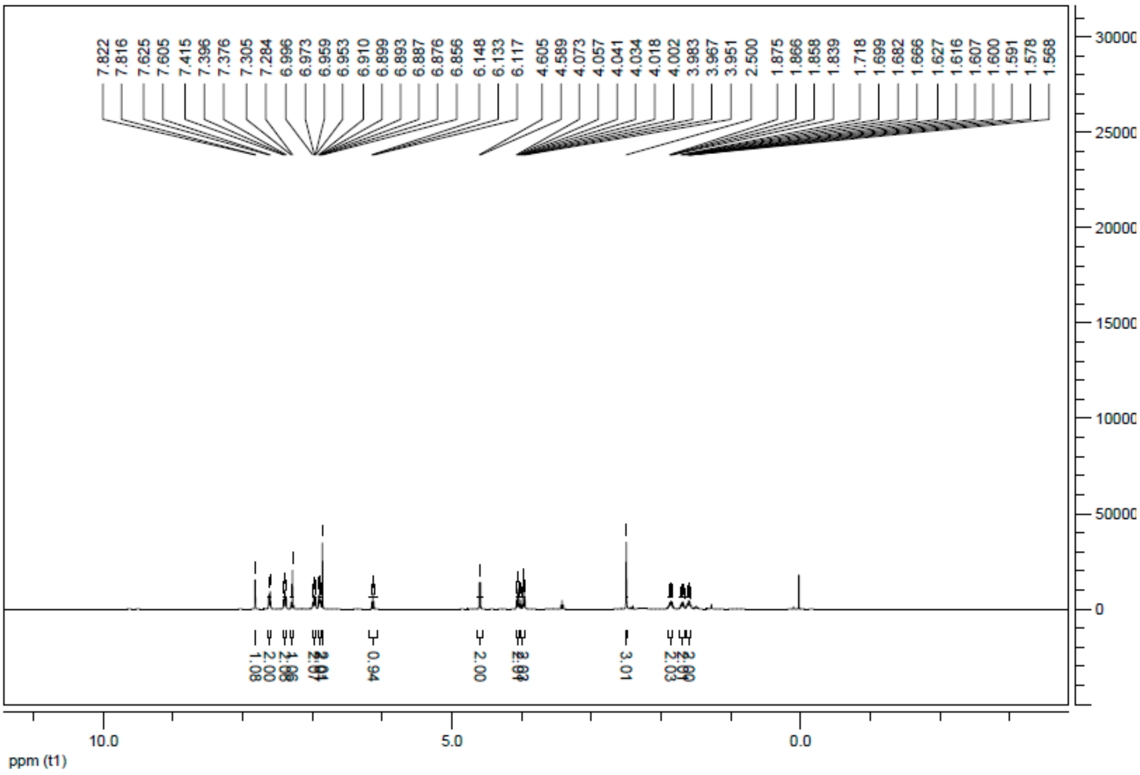

Figure S35. ^{1}H-NMR of compound **7r** (400 MHz, $CDCl_3$).

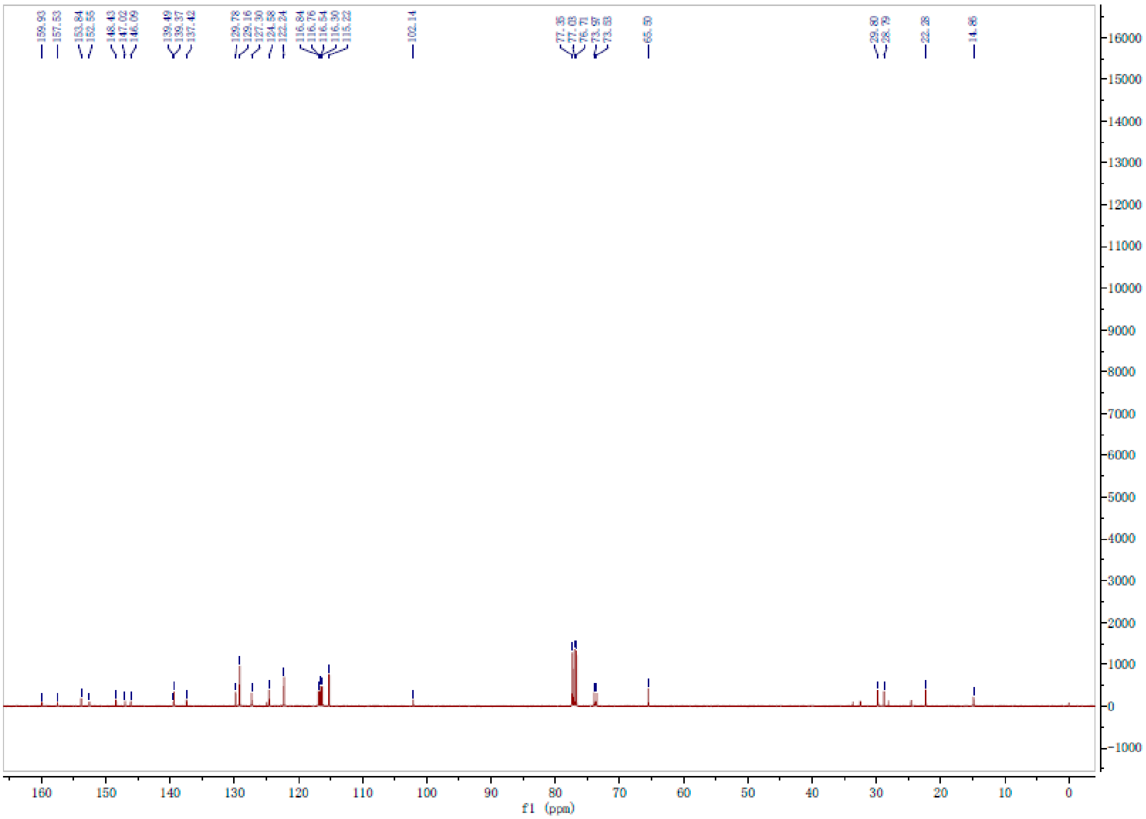

Figure S36. ^{13}C-NMR of compound **7r** (100 MHz, $CDCl_3$).
